# Biomarker Concentrations and Their Temporal Changes in Patients With Myocardial Infarction and Nonobstructive Compared With Obstructive Coronary Arteries: Results From the PLATO Trial

**DOI:** 10.1161/JAHA.122.027466

**Published:** 2022-12-24

**Authors:** Marcus Hjort, Kai M. Eggers, Tatevik Ghukasyan Lakic, Johan Lindbäck, Andrzej Budaj, Jan H. Cornel, Evangelos Giannitsis, Hugo A. Katus, Agneta Siegbahn, Robert F. Storey, Richard C. Becker, Lars Wallentin, Bertil Lindahl, the PLATO trial investigators*

**Affiliations:** ^1^ Department of Medical Sciences Uppsala University Uppsala Sweden; ^2^ Uppsala Clinical Research Center Uppsala University Uppsala Sweden; ^3^ Department of Cardiology, Centre of Postgraduate Medical Education Grochowski Hospital Warsaw Poland; ^4^ Department of Cardiology, Northwest Clinics Alkmaar, and Radboud University Medical Center Nijmegen The Netherlands; ^5^ Department of Medicine III University of Heidelberg Heidelberg Germany; ^6^ Department of Infection, Immunity and Cardiovascular Disease University of Sheffield Sheffield United Kingdom; ^7^ Division of Cardiovascular Health and Diseases University of Cincinnati Heart, Lung & Vascular Institute Cincinnati OH

**Keywords:** biomarkers, myocardial infarction, myocardial infarction with nonobstructive coronary arteries, pathophysiology, Biomarkers, Proteomics, Mechanisms, Myocardial Infarction, Inflammatory Heart Disease

## Abstract

**Background:**

The pathobiology of myocardial infarction (MI) with nonobstructive coronary arteries (MINOCA) is often uncertain. Investigating biomarker concentrations and their changes may offer novel pathophysiological insights.

**Methods and Results:**

In this post hoc study of the PLATO (Platelet Inhibition and Patient Outcomes) trial, concentrations of hs‐cTnT (high‐sensitivity cardiac troponin T), NT‐proBNP (N‐terminal pro‐B‐type natriuretic peptide), hs‐CRP (high‐sensitivity C‐reactive protein), and GDF‐15 (growth differentiation factor 15) were measured in patients with MINOCA at baseline (n=554) and at 1‐month follow‐up (n=107). For comparisons, biomarkers were also measured in patients with MI with obstructive (stenosis ≥50%) coronary artery disease (baseline: n=11 106; follow‐up: n=2755]). Adjusted linear regression models were used to compare concentrations and their short‐ and long‐term changes. The adjusted geometric mean ratios (GMRs) in patients with MINOCA (median age, 61 years; 50.4% women) indicated lower hs‐cTnT (GMR, 0.77 [95% CI, 0.68–0.88]) but higher hs‐CRP (GMR, 1.21 [95% CI, 1.08–1.37]) and GDF‐15 concentrations (GMR, 1.06 [95% CI, 1.02–1.11]) at baseline compared with patients with MI with obstructive coronary artery disease, whereas NT‐proBNP concentrations were similar. Temporal decreases in hs‐cTnT, NT‐proBNP, and hs‐CRP concentrations until 1‐month follow‐up were more pronounced in patients with MINOCA. At follow‐up, patients with MINOCA had lower concentrations of hs‐cTnT (GMR, 0.71 [95% CI, 0.60–0.84]), NT‐proBNP (GMR, 0.45 [95% CI, 0.36–0.56]), and hs‐CRP (GMR, 0.68 [95% CI, 0.53–0.86]). One‐month GDF‐15 concentrations were similar between both groups with MI.

**Conclusions:**

Biomarker concentrations suggest greater initial inflammatory activity, similar degree of myocardial dysfunction, and less pronounced myocardial injury during the acute phase of MINOCA compared with MI with obstructive coronary artery disease but also faster myocardial recovery.

**Registration:**

URL: http://www.clinicaltrials.gov; Unique identifier: NCT00391872.

**Nonstandard Abbreviations and Acronyms**
GDF‐15: growth differentiation factor 15
GMR: geometric mean ratio
hs‐cTnT: high‐sensitivity cardiac troponin T
MINOCA: myocardial infarction with nonobstructive coronary arteries
PLATO: Platelet Inhibition and Patient Outcomes
SMINC: Stockholm Myocardial Infarction With Normal Coronaries

**Clinical Perspective**

**What Is New?**

- The present study is the first to examine temporal changes in established biomarkers of inflammation and myocardial damage/dysfunction in myocardial infarction (MI) with nonobstructive coronary arteries.
- Biomarkers indicate differences in pathobiology between patients with MI with nonobstructive coronary arteries and MI with obstructive coronary artery disease, with greater initial inflammatory activity, similar degree of myocardial dysfunction, and less pronounced myocardial damage during the acute phase of MINOCA.
- Also, there were a more transient myocardial affection and faster recovery in patients with MINOCA compared with MI‐CAD.

**What Are the Clinical Implications?**

- Our results aid in understanding the pathophysiology of MI with nonobstructive coronary arteries and highlight processes that may be further explored on patient management.

Around 5% to 10%^1^, ^2^, ^3^ of patients with acute myocardial infarction (MI) present as MI with nonobstructive (normal or <50% stenosis) coronary arteries (MINOCA).^1^, ^2^ Patients with MINOCA have a 1‐year mortality of 3.4% in a meta‐analysis^4^ from 2021, and no comprehensive treatment guidelines exist.^1^, ^2^ In contrast to MI with obstructive coronary artery disease (MI‐CAD), patients with MINOCA tend to have fewer traditional risk factors, are often younger, often present with non–ST‐segment elevation instead of ST‐segment elevation, and around half of them are women.^2^, ^4^ The underlying pathophysiology of MINOCA is heterogeneous and often unknown.^1^, ^2^, ^5^, ^6^ Proposed mechanisms are coronary spasm, plaque disruption, thrombus that rapidly has dissolved, dissection, microvascular dysfunction, inflammation, and imbalance in myocardial oxygen supply/demand.^1^, ^2^, ^3^, ^5^, ^7^, ^8^, ^9^ Hence, MINOCA is considered a working diagnosis, and careful individualized patient work‐up is advised to find a treatable mechanism.

Studies investigating the mechanisms behind MINOCA have relied on assessment of clinical risk factors, noninvasive myocardial imaging, and invasive coronary visualization and functional testing.^1^, ^2^, ^3^ Biomarkers may also be used to reflect different pathological processes in MINOCA (ie, of myocardial dysfunction/injury, inflammation, atherogenesis/atherosclerosis, and hemostasis) and have been studied to some extent in MINOCA.^8^, ^9^, ^10^, ^11^, ^12^, ^13^, ^14^, ^15^, ^16^ However, most of these studies have investigated one or a few biomarkers collected at a single time point during the index hospitalization.^10^, ^11^, ^12^, ^13^, ^14^, ^15^, ^16^ Information on how conventional biomarker concentrations change over the first few weeks after an episode of MINOCA might aid in better understanding the pathophysiological mechanisms.

The aim of the present analysis was to explore concentrations and changes of biomarkers of myocardial damage (hs‐cTnT [high‐sensitivity cardiac troponin T]), myocardial dysfunction (NT‐proBNP [N‐terminal pro‐B‐type natriuretic peptide]), inflammation (hs‐CRP [high‐sensitivity C‐reactive protein], GDF‐15 [growth differentiation factor 15]), and oxidative stress and hypoxia (GDF‐15)^17^, ^18^, ^19^ in patients with MINOCA, both in the acute phase of the disease and up until 1 month thereafter. For comparative purposes, these metrics were also studied in patients with MI‐CAD.

## METHODS

The data set analyzed in this study is not publicly available because of ethical restrictions. However, access can be made available at Uppsala Clinical Research Center (Uppsala, Sweden) upon reasonable request and under the provision that the data are accessed on site and do not leave Uppsala University.

### Study Population

**Figure 1:**
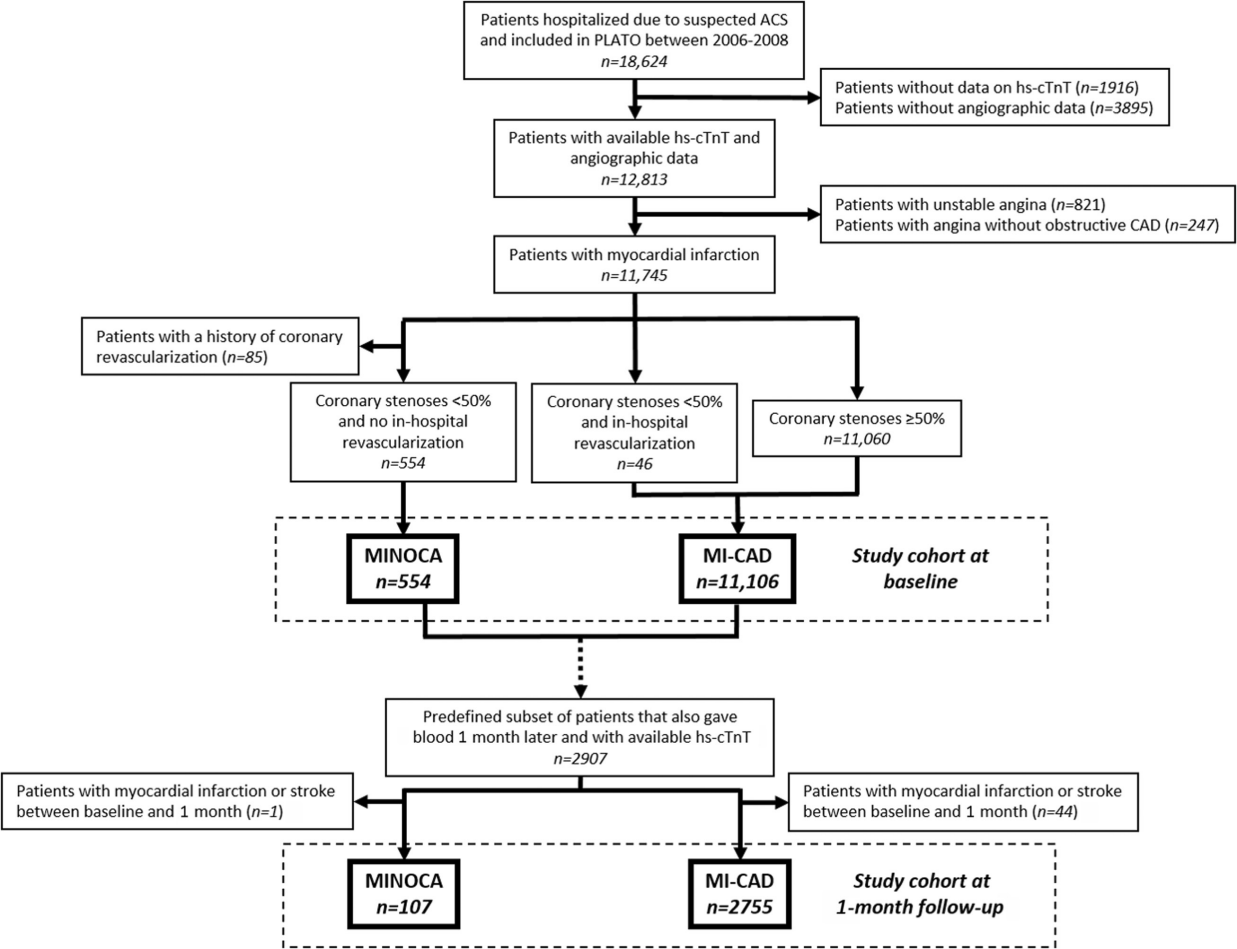
Inclusion of patients into groups with MINOCA and MI‐CAD. ACS indicates acute coronary syndrome; CAD, coronary artery disease; hs‐cTnT, high‐sensitivity cardiac troponin T; MI‐CAD, myocardial infarction with obstructive coronary artery disease; MINOCA, myocardial infarction with nonobstructive coronary arteries; and PLATO, Platelet Inhibition and Patient Outcomes.

This is an observational post hoc study of patients included in the PLATO (Platelet Inhibition and Patient Outcomes) trial (ClinicalTrials.gov identifier: NCT00391872) between October 2006 and July 2008. The PLATO trial was an international, multicenter, double‐blind, randomized control trial comparing ticagrelor and clopidogrel in addition to aspirin for the prevention of cardiovascular events in 18 624 patients hospitalized because of a spontaneous acute coronary syndrome.^20^, ^21^ From these patients, blood samples for analyses of circulating biomarkers were collected at the time of randomization, henceforth referred to as blood sampling at baseline. In addition, blood samples at 1 month later were analyzed in patients at selected study sites that were part of the prespecified 1‐month follow‐up biomarker substudy (around 4000 patients) of the PLATO trial program.^20^ Because biomarker concentrations might be affected by intercurrent ischemic stroke or new MI, we excluded such patients from the investigation of 1‐month biomarker results. Unfortunately, no information on potential complications to revascularization was registered in the PLATO trial and was therefore not accounted for. Figure 1 illustrates the inclusion of patients into groups with MINOCA and MI‐CAD.

The PLATO trial and the current study were conducted in adherence with the Declaration of Helsinki and were approved by ethical review boards. All patients provided written informed consent to participate and received routine medical care with the exception of randomized treatment (ticagrelor or clopidogrel).

### Definitions

The final diagnosis of MI together with the presence of ST‐segment–elevation MI (STEMI) or non–ST‐segment–elevation MI (NSTEMI) was determined at discharge by the investigators at each trial site. There was no formal adjudication of the index diagnoses. Patients were deemed to have MINOCA when diagnosed as MI but without a significant (ie, <50%) coronary stenosis in any vessel at coronary angiography and without coronary revascularization before or during the index hospitalization. The reason for excluding patients with prior revascularization from the cohort with MINOCA was to dismiss patients with previous manifest obstructive coronary artery disease. Patients with MI with a significant (ie, ≥50%) coronary stenosis at coronary angiography in at least 1 vessel were considered as having MI‐CAD. A small number of patients classified as having nonobstructive stenoses underwent coronary revascularization, although percutaneous coronary intervention of MINOCA lesions is not encouraged in the consensus documents on MINOCA of the European Society of Cardiology^1^ and the American Heart Association.^2^ These patients were, therefore, regarded as having MI‐CAD.

### Biomarker Analysis

Plasma was collected by venous puncture at baseline (a median of 15.1 and 8.9 hours from onset of symptoms for patients with MINOCA and MI‐CAD, respectively) and after 1 month in a subset of paired patients and stored in aliquots in −70 °C until analysis. Baseline blood samples were obtained in all patients before coronary angiography. Biomarker concentrations were centrally measured at the Uppsala Clinical Research Center Laboratory (Uppsala, Sweden): hs‐cTnT, NT‐proBNP, and GDF‐15 were analyzed on Cobas Analytics Immunoanalyzers (Roche Diagnostics); and hs‐CRP was analyzed on the Architect platform (Abbott Diagnostics). All analyses were done according to the instructions of the assay manufacturers and have been described in detail previously.^22^

### Statistical Analysis

Categorical variables were reported as frequencies and percentages, and continuous variables were reported as medians with interquartile ranges. Distributions of biomarker concentrations were displayed in empirical cumulative distribution function plots.

Differences in biomarker concentration distributions between MINOCA and MI‐CAD at baseline and at 1 month later were investigated using Mann‐Whitney tests. To study these differences adjusting for potential confounders, we performed linear regression analyses using ln‐transformed concentrations of hs‐cTnT, NT‐proBNP, hs‐CRP, and GDF‐15 as response variables. The concentrations are presented as geometric means and were calculated using the antilogs of the model‐adjusted means of the ln‐transformed data; thus, the comparisons between MINOCA and MI‐CAD are presented as adjusted geometric mean ratios. A geometric mean ratio >1 corresponds to a higher biomarker concentration in MINOCA, and a geometric mean ratio <1 corresponds to a higher biomarker concentration in MI‐CAD. The analyses were adjusted in 2 models. Model 1 was unadjusted at baseline, whereas at 1‐month follow‐up, it was adjusted for randomized treatment (clopidogrel versus ticagrelor) and corresponding biomarker concentration at baseline. Model 2 was additionally adjusted for baseline clinical characteristics (age, sex, body mass index, current smoking, hypertension, dyslipidemia [medical history or lipid treatment at admission], diabetes, chronic kidney disease [medical history], MI type [STEMI/NSTEMI], previous MI, previous ischemic stroke, previous heart failure, and peripheral vascular disease). Additional adjustment was made at baseline for hours from the onset of symptoms to blood sampling, and at 1‐month follow‐up for revascularization during index hospitalization. A further analysis was done with adjustment for medications at follow‐up (antiplatelets, P2Y_12_ inhibitors, β‐blockers, renin‐angiotensin‐aldosterone system inhibitors, statins, and calcium channel blockers).

To visualize temporal biomarker concentration changes in MINOCA and MI‐CAD during hospital stay, linear regression models including time from symptom onset, MINOCA/MI‐CAD classification, and MI type (STEMI/NSTEMI), as well as all pairwise interactions between the 3, were fitted. Time was modeled as a restricted cubic spline, with 4 knots placed at the 5th, 35th, 65th, and 95th sample percentiles, to allow for nonlinear associations (for details, see Data S1). The spline model was adjusted for the same variables as in model 2. Also, the global hypothesis of no 2‐way interaction (between any pair of: time from symptom onset, MINOCA/MI‐CAD status, and STEMI/NSTEMI status) was tested.

Differences between MINOCA and MI‐CAD in delta changes of biomarker concentrations from baseline to 1 month later were assessed using Mann‐Whitney tests.

There were a few missing biomarker values that were not accounted for in the statistical analyses. In all tests, a statement of statistical significance implies a 2‐sided *P*<0.05. The software packages R 4.1.1 (R Foundation for Statistical Computing, Vienna, Austria) and SAS 9.4 (SAS Institute Inc, Cary, NC) were used for the statistical analyses.

## RESULTS

Biomarker results were available at baseline in 554 patients with MINOCA and 11 106 patients with MI‐CAD; at 1‐month follow‐up, biomarker results were available in 107 patients with MINOCA and 2755 patients with MI‐CAD (Figure 1). Table 1 shows baseline data on demography, risk factors, medical history, MI type (STEMI/NSTEMI), in‐hospital revascularization, and medications for both the total cohort and patients with available 1‐month biomarker results. Medications at blood sampling at 1‐month follow‐up are displayed in Table S1. Compared with patients with MI‐CAD, those with MINOCA were more often women, had fewer cardiovascular risk factors, and less often had angina pectoris, previous MI, peripheral arterial disease, and chronic kidney disease.

**Table 1:** Baseline Characteristics in Patients With MINOCA and MI‐CAD

Characteristic	Total cohort at baseline		Follow‐up cohort at baseline	
	MINOCA (n=554)	MI‐CAD (n=11 106)	MINOCA (n=107)	MI‐CAD (n=2755)
Demography				
Age, y	61 (51–70)	61 (53–70)	61 (51–69)	61 (53–69)
Women	279 (50.4)	2643 (23.8)	56 (52.3)	691 (25.1)
BMI, kg/m^2^	27.2 (24.2–30.5)	27.4 (24.8–30.4)	27.7 (24.5–30.4)	27.6 (25.1–30.5)
Risk factors				
Current smoking	171 (30.9)	4446 (40.0)	28 (26.2)	1149 (41.7)
Hypertension	336 (60.6)	6921 (62.3)	71 (66.4)	1708 (62.0)
Dyslipidemia*	211 (38.1)	5224 (47.0)	37 (34.6)	1089 (39.5)
Diabetes	100 (18.1)	2645 (23.8)	14 (13.1)	562 (20.4)
eGFR, mL/min per 1.73 m^2^ †	108 (82–120)	110 (83–120)	94 (81–119)	92 (71–116)
History				
Angina pectoris	142 (25.6)	4316 (38.9)	26 (24.3)	1122 (40.7)
Previous MI	35 (6.3)	1903 (17.1)	8 (7.5)	436 (15.8)
Previous PCI	…	1406 (12.7)	…	279 (10.1)
Previous CABG	…	588 (5.3)	…	116 (4.2)
Previous heart failure	22 (4.0)	357 (3.2)	3 (2.8)	88 (3.2)
Previous ischemic stroke	22 (4.0)	331 (3.0)	4 (3.7)	71 (2.6)
Peripheral arterial disease	15 (2.7)	627 (5.6)	3 (2.8)	143 (5.2)
Chronic kidney disease	13 (2.3)	405 (3.6)	1 (0.9)	86 (3.1)
MI type				
STEMI	142 (25.6)	5489 (49.4)	29 (27.1)	1598 (58.0)
NSTEMI	412 (74.4)	5617 (50.6)	78 (72.9)	1157 (42.0)
In‐hospital revascularization				
PCI	…	9143 (82.3)	…	2437 (88.5)
CABG	…	689 (6.2)	…	85 (3.1)
Medications at blood sampling				
Aspirin	521 (94.0)	10 616 (95.6)	103 (96.3)	2679 (97.2)
Clopidogrel	180 (32.5)	4349 (39.2)	33 (30.8)	720 (26.1)
β‐Blockers	416 (75.1)	8235 (74.1)	86 (80.4)	2061 (74.8)
RAAS inhibitors	322 (58.1)	6849 (61.7)	65 (60.7)	1861 (67.5)
Statins	409 (73.8)	8984 (80.9)	83 (77.6)	2337 (84.8)
Calcium inhibitors	67 (12.1)	1519 (13.7)	14 (13.1)	381 (13.8)
Diuretics	110 (19.9)	2288 (20.6)	17 (15.9)	571 (20.7)

Data presented as numbers (with percentages) for categorical variables or medians (with interquartile ranges) for continuous variables. Information on clinical characteristics and in‐hospital revascularization was collected at the index hospitalization. Medications were considered at the time of blood sampling at baseline. BMI indicates body mass index; CABG, coronary artery bypass grafting; eGFR, estimated glomerular filtration rate; MI, myocardial infarction; MI‐CAD, MI with obstructive coronary artery disease; MINOCA, MI with nonobstructive coronary arteries; NSTEMI, non–ST‐segment–elevation MI; PCI, percutaneous coronary intervention; RAAS, renin‐angiotensin‐aldosterone system; and STEMI, ST‐segment–elevation MI.

* On the basis of medical history or lipid treatment at admission.

^†^ On the basis of cystatin C. Missing: n=17 patients with MINOCA and n=300 patients with MI‐CAD in the baseline cohort; n=28 patients with MI‐CAD in the follow‐up cohort.

**Figure 2:**
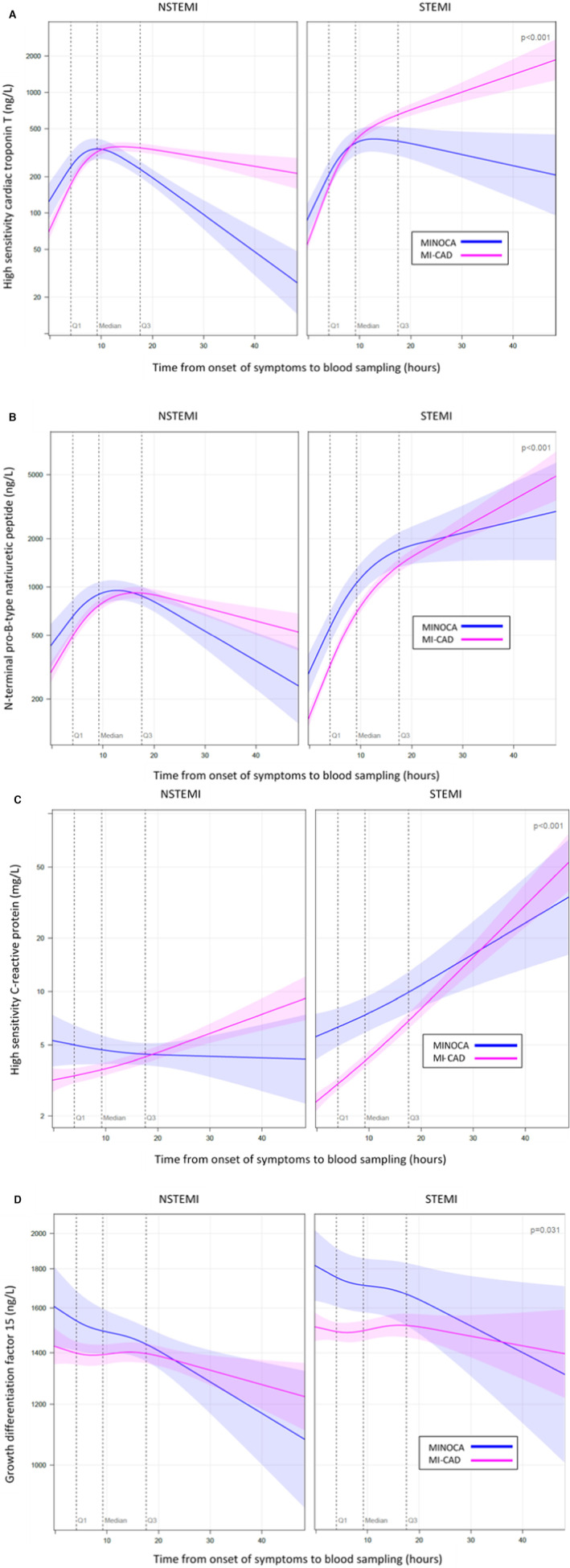
Adjusted spline models illustrating the association of mean biomarker concentrations with time in patients with MINOCA and MI‐CAD divided on STEMI or NSTEMI status. **A**, Hs‐cTnT (high‐sensitivity cardiac troponin T). **B**, NT‐proBNP (N‐terminal pro‐B‐type natriuretic peptide). **C**, Hs‐CRP (high‐sensitivity C‐reactive protein). **D**, GDF‐15 (growth differential factor 15). Time was modeled as a restricted cubic spline and included MINOCA/MI‐CAD classification and myocardial infarction (MI) type (STEMI/NSTEMI) as well as all pairwise interactions among these 3 variables. *P* values are from a global test of the hypothesis of no 2‐way interaction between any pair of the 3 variables. The shaded colored area represents pointwise 95% CIs. The model is adjusted for baseline clinical characteristics (age, sex, body mass index, current smoking, hypertension, dyslipidemia [medical history and/or lipid treatment at admission], diabetes, chronic kidney disease [medical history], previous MI, previous ischemic stroke, previous heart failure, and peripheral vascular disease). MI‐CAD indicates myocardial infarction with obstructive coronary artery disease; MINOCA, myocardial infarction with nonobstructive coronary arteries; NSTEMI, non–ST‐segment–elevation myocardial infarction; and STEMI, ST‐segment–elevation myocardial infarction.

Overall, the time from onset of symptoms to blood sampling at baseline was 15.1 (8.1–21.3) hours in patients with MINOCA and 8.9 (3.9–17.3) hours in those with MI‐CAD. Blood samples were collected closer to the onset of symptoms in patients with STEMI compared with NSTEMI, in both the MINOCA and the MI‐CAD groups (Table 2). In addition, the global test of interaction between any pair of time from symptom onset, MINOCA/MI‐CAD classification, and MI type (STEMI/NSTEMI) was statistically significant for each independent biomarker in the spline models (Figure 2).

**Table 2:** Time From Symptom Onset to Blood Sampling at Baseline in the Total Cohort and Concentrations of Biomarkers Stratified by MINOCA or MI‐CAD and STEMI or NSTEMI

Variable	MINOCA (n=554)	MI‐CAD (n=11 106)
Time from symptom onset to blood sampling, h		
STEMI	7.5 (3.9–14.9)	4.7 (2.8–9.6)
NSTEMI	17.5 (11.3–22.4)	14.8 (8.2–20.3)
All	15.1 (8.1–21.3)	8.9 (3.9–17.3)
Time from symptom onset to angiography, h		
STEMI	9.1 (4.7–22.7)	5.1 (3.0–10.9)
NSTEMI	27.1 (17.8–53.4)	22.9 (12.7–43.7)
All	23.7 (11.5–46.3)	12.0 (4.5–26.0)
hs‐cTnT, ng/L		
STEMI	320 (94–730)	200 (59–735)
NSTEMI	220 (81–589)	278 (102–733)
All	241 (86–630)	242 (78–735)
NT‐proBNP, ng/L		
STEMI	546 (186–1553)	282 (84–1024)
NSTEMI	586 (204–1911)	605 (252–1359)
All	572 (199–1823)	451 (149–1218)
hs‐CRP, mg/L		
STEMI	6.1 (2.6–27.5)	3.3 (1.5–8.4)
NSTEMI	4.4 (1.7–12.0)	3.9 (1.8–9.5)
All	4.8 (1.9–14.0)	3.6 (1.6–9.0)
GDF‐15, ng/L		
STEMI	1554 (1024–2517)	1509 (1128–2148)
NSTEMI	1439 (1113–2030)	1515 (1130–2136)
All	1454 (1075–2099)	1513 (1129–2144)

Data presented as medians (with interquartile ranges). GDF‐15 indicates growth differentiation factor 15; hs‐CRP, high‐sensitivity C‐reactive protein; hs‐cTnT, high‐sensitivity cardiac troponin T; MI‐CAD, myocardial infarction with obstructive coronary artery disease; MINOCA, myocardial infarction with nonobstructive coronary arteries; NSTEMI, non–ST‐segment–elevation myocardial infarction; NT‐proBNP, N‐terminal pro‐B‐type natriuretic peptide; and STEMI, ST‐segment–elevation myocardial infarction.

### Biomarker Concentrations at Baseline and Short‐Term Changes

Baseline concentrations of hs‐cTnT, NT‐proBNP, hs‐CRP, and GDF‐15 in the total cohort of patients with MINOCA and MI‐CAD are presented in Table 3 and Figure S1, respectively.

**Table 3:** Biomarker Concentrations and Multiple Linear Regression Results in the Total Cohort at Baseline

Biomarker	Total cohort at baseline					
	Biological function	MINOCA (n=554)	MI‐CAD (n=11 106)	Missing (MINOCA/MI‐CAD)	Multiple linear regression	
		Biomarker concentration			Geometric mean ratio (95% CI)	
					Model 1	Model 2
hs‐cTnT, ng/L	Myocardial damage	241 (86–630)	242 (78–735)	0/0	0.93 (0.82–1.06)	0.77 (0.68–0.88)*
NT‐proBNP, ng/L	Myocardial dysfunction	572 (199–1823)	451 (149–1218)	0/1	1.43 (1.26–1.62)*	1.10 (0.99–1.23)
hs‐CRP, mg/L	Proinflammation	4.8 (1.9–14.0)	3.6 (1.6–9.0)	17/298	1.32 (1.17–1.48)*	1.21 (1.08–1.37)*
GDF‐15, ng/L	Inflammation Oxidative stress Hypoxia	1454 (1075–2099)	1513 (1129–2144)	0/1	0.98 (0.93–1.02)	1.06 (1.02–1.11)*

Biomarker data presented as medians (with interquartile ranges). Model 1: unadjusted. Model 2: adjusted for baseline clinical characteristics (age, sex, body mass index, current smoking, hypertension, dyslipidemia [medical history and/or lipid treatment at admission], diabetes, chronic kidney disease [medical history], myocardial infarction [MI] type [ST‐segment–elevation MI/non–ST‐segment–elevation MI], previous MI, previous ischemic stroke, previous heart failure, peripheral vascular disease, and hours from the onset of symptoms to blood sampling). GDF‐15 indicates growth differentiation factor 15; hs‐CRP, high‐sensitivity C‐reactive protein; hs‐cTnT, high‐sensitivity cardiac troponin T; MI‐CAD, MI with obstructive coronary artery disease; MINOCA, MI with nonobstructive coronary arteries; and NT‐proBNP, N‐terminal pro‐B‐type natriuretic peptide.

* Statistically significant values.

Crude hs‐cTnT concentrations were similar between patients with MINOCA and MI‐CAD (Table 3; model 1). Overall, mean hs‐cTnT concentrations peaked at 10 hours from the onset of symptoms in NSTEMI, with a more rapid decrease seen in MINOCA compared with MI‐CAD (Figure 2A). Mean hs‐cTnT concentrations continued to increase in patients with MI‐CAD with STEMI until at least 50 hours from the symptom onset, which was in contrast to patients with MINOCA with STEMI. On multivariable adjustment, the geometric mean ratio of hs‐cTnT was statistically significantly <1 (Table 3; model 2). This indicates lower hs‐cTnT concentrations in MINOCA when considering clinical characteristics, including time from symptom onset and MI type (STEMI/NSTEMI).

Patients with MINOCA had higher crude NT‐proBNP concentrations compared with those with MI‐CAD (Table 3; model 1), although concentrations were instead similar following adjustments (model 2). Figure 2B illustrates that mean NT‐proBNP concentrations peaked at 10 to 20 hours in patients with NSTEMI, with a more rapid decrease in those with MINOCA. Among patients with STEMI, mean NT‐proBNP concentrations increased steadily and similarly between patients with MINOCA and MI‐CAD.

hs‐CRP concentrations were higher in patients with MINOCA compared with MI‐CAD in both crude and adjusted analyses (Table 3; models 1 and 2). Regardless of the presence of STEMI or NSTEMI, mean hs‐CRP concentrations were higher in patients with MINOCA during the first 20 hours (Figure 2C). Mean hs‐CRP changes differed among patients with NSTEMI, with constant or slightly decreasing concentrations in MINOCA; however, there were increasing concentrations in patients with MI‐CAD over time. In patients with STEMI, in contrast, mean hs‐CRP concentrations progressively increased in both those with MINOCA and MI‐CAD up until 50 hours from the index event.

The crude GDF‐15 concentrations were similar between patients with MINOCA and MI‐CAD (Table 3; model 1) but marginally higher in patients with MINOCA after adjustments (model 2). Mean GDF‐15 concentrations were higher during the first 35 hours in patients with MINOCA and STEMI, although only slightly higher during the first 25 hours in those with NSTEMI (Figure 2D). Furthermore, mean concentrations after these first hours were then similar and decreased in patients with MINOCA and MI‐CAD regardless of the presence of STEMI or NSTEMI.

### Biomarker Concentrations at 1‐Month Follow‐Up and Long‐Term Changes Over Time

**Figure 3:**
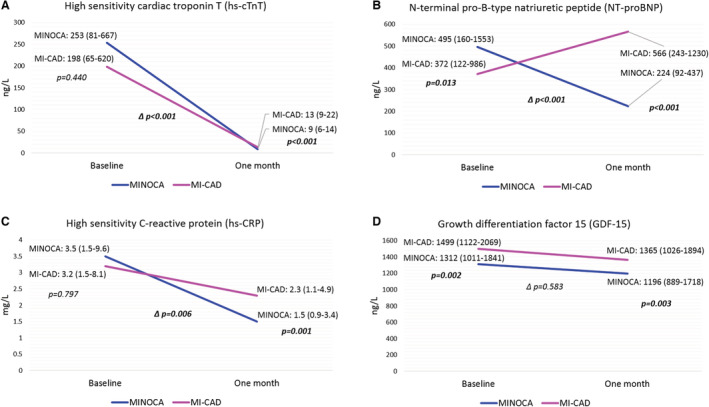
Temporal changes in biomarker concentrations from baseline to 1 month later in the follow‐up cohort of patients with MINOCA and MI‐CAD. **A**, hs‐cTnT. **B**, NT‐proBNP . **C**, hs‐CRP. **D**, GDF‐15. Biomarker data presented as medians (with interquartile ranges). *P* values indicate unadjusted differences in concentrations between patients with MINOCA and MI‐CAD at baseline and at 1 month later. ∆*P* values indicate unadjusted differences between patients with MINOCA and MI‐CAD in absolute changes of concentrations from baseline to 1 month later. Bold *P* values indicate statistical significance. GDF indicates growth differentiation factor; hs‐CRP, high‐sensitivity C‐reactive protein; hs‐cTnT, high‐sensitivity cardiac troponin T; MI‐CAD, myocardial infarction with obstructive coronary artery disease; MINOCA, myocardial infarction with nonobstructive coronary arteries; and NT‐proBNP, N‐terminal pro‐B‐type natriuretic peptide.

One‐month biomarker concentrations in the follow‐up cohort are given in Table 4 and Figure S2. Hs‐cTnT concentrations decreased in patients with both MINOCA and MI‐CAD from baseline to 1 month later (Figure 3A), with lower concentrations in patients with MINOCA at follow‐up, according to adjusted analyses (Table 4). For NT‐proBNP, concentrations decreased in patients with MINOCA but increased in patients with MI‐CAD (Figure 3B). Accordingly, the latter group had higher concentrations at follow‐up. Hs‐CRP concentrations decreased in both patients with MINOCA and MI‐CAD (Figure 3C). The degree of decrease was more pronounced in patients with MINOCA, resulting in lower concentrations in this cohort. There was a slight decrease in GDF‐15 concentrations that was similar between patients with MINOCA and MI‐CAD (Figure 3D), and, conditional on the baseline concentrations, adjusted 1‐month concentrations were also similar in patients with MINOCA and MI‐CAD (Table 4). Additional adjustment for medications at 1‐month follow‐up (antiplatelets, P2Y_12_ inhibitors, β‐blockers, renin‐angiotensin‐aldosterone system inhibitors, statins, and calcium channel blockers) did not alter the results (data not shown).

**Table 4:** Biomarker Concentrations and Multiple Linear Regression Results in the Follow‐Up Cohort at 1 Month After the MI

Biomarker	Follow‐up cohort at 1 mo					
	Biological function	MINOCA (n=107)	MI‐CAD (n=2755)	Missing (MINOCA/MI‐CAD)	Multiple linear regression	
		Biomarker concentration			Geometric mean ratio (95% CI)	
					Model 1	Model 2
hs‐cTnT, ng/L	Myocardial damage	9 (6–14)	13 (9–22)	0/0	0.65 (0.56–0.76)*	0.71 (0.60–0.84)*
NT‐proBNP, ng/L	Myocardial dysfunction	224 (92–437)	566 (243–1230)	0/0	0.36 (0.29–0.44)*	0.45 (0.36–0.56)*
hs‐CRP, mg/L	Proinflammation	1.5 (0.9–3.4)	2.3 (1.1–4.9)	0/27	0.67 (0.55–0.82)*	0.68 (0.53–0.86)*
GDF‐15, ng/L	Inflammation Oxidative stress Hypoxia	1196 (889–1718)	1366 (1027–1891)	0/0	0.97 (0.91–1.05)	0.95 (0.88–1.02)

Biomarker data presented as medians (with interquartile ranges). Model 1: adjusted for randomized treatment (clopidogrel vs ticagrelor) and corresponding biomarker concentration at baseline. Model 2: additionally adjusted for baseline clinical characteristics (age, sex, body mass index, current smoking, hypertension, dyslipidemia [medical history and/or lipid treatment at admission], diabetes, chronic kidney disease [medical history], MI type [ST‐segment–elevation MI/non–ST‐segment–elevation MI], previous MI, previous ischemic stroke, previous heart failure, peripheral vascular disease, and revascularization during index hospitalization). GDF‐15 indicates growth differentiation factor 15; hs‐CRP, high‐sensitivity C‐reactive protein; hs‐cTnT, high‐sensitivity cardiac troponin T; MI, myocardial infarction; MI‐CAD, MI with obstructive coronary artery disease; MINOCA, MI with nonobstructive coronary arteries; and NT‐proBNP, N‐terminal pro‐B‐type natriuretic peptide.

* Statistically significant values.

## DISCUSSION

This is the first study examining temporal changes in established biomarkers of inflammation and myocardial damage and dysfunction in patients with MINOCA compared with MI‐CAD. Biomarkers were measured in the acute phase of the index MI and 1 month later in a follow‐up cohort, and careful consideration was taken of variables that potentially influence biomarker concentrations and dynamics. Our results indicate differences in the pathobiology between the MI groups, with more pronounced initial inflammatory activity and smaller myocardial injury during the acute phase of MINOCA and a more transient myocardial affection and faster recovery compared with MI‐CAD.

### Differences in Biomarker Concentrations in the Acute Phase of MI

In the acute phase, the adjusted linear model showed higher hs‐cTnT concentrations in patients with MI‐CAD compared with MINOCA, indicating a greater degree of myocardial injury in those with MI‐CAD. Indeed, numerically^8^, ^12^ and significantly^10^, ^11^, ^23^ higher concentrations of cardiac troponins in patients with MI‐CAD compared with MINOCA during the index hospitalization of MI have previously been demonstrated. There was also indication of a more transient myocardial injury in patients with MINOCA, as mean hs‐cTnT concentrations were declining already after 10 hours. This difference in hs‐cTnT concentrations between the MINOCA and MI‐CAD groups could have been attributed to the higher prevalence of STEMI in patients with MI‐CAD, because STEMI results in a larger infarcted myocardial area than NSTEMI.^24^ It could also in part have been attributable to variations in the timing of blood sampling from symptom onset between patients with STEMI and NSTEMI, given the early release kinetics of cardiac troponins in MI.^25^ However, the difference in hs‐cTnT concentrations between patients with MINOCA and MI‐CAD persisted in the fully adjusted model, which considered both timing to blood sampling and the presence of STEMI or NSTEMI.

NT‐proBNP concentrations in patients with MINOCA were similar to patients with MI‐CAD, except for patients with NSTEMI, in whom a more rapid decline was seen after the initial 10 to 20 hours. This corresponds with previous data^10^ and indicates less severe and more transient myocardial dysfunction in patients with MINOCA without ST‐segment elevation. In patients with STEMI, in contrast, NT‐proBNP followed a similar pattern in patients with MINOCA and MI‐CAD, with increasing mean concentrations over time.

Hs‐CRP concentrations were overall higher in patients with MINOCA compared with MI‐CAD. This may be attributable to a proinflammatory disposition in patients with MINOCA.^8^, ^9^, ^26^ The fact that the spline plots indicated higher mean hs‐CRP concentrations in patients with MINOCA during the first 20 hours from onset of symptoms appears to support this notion. MINOCA may be linked to inflammation through different pathways. Systemic inflammation in general and pericoronary inflammation^27^ in particular may trigger coronary spasm,^28^ microvascular dysfunction,^29^ and destabilization and rupture of an angiographically nonsignificant atherosclerotic plaque. However, during the acute phase of MI, inflammatory mediators, like CRP and interleukin‐6, are mainly associated with the extent of myocardial injury, secondary necrosis, and left ventricular remodeling.^30^ This is reflected by increasing hs‐CRP concentrations in patients with MI‐CAD but also those with MINOCA who had STEMI. The mechanisms behind these differences in hs‐CRP concentrations in patients with MINOCA compared with MI‐CAD is yet unclear, and caution is required when interpreting our data, given the small size of this patient subset. This notion is further supported by conflicting results from other studies^10^, ^12^, ^16^ where hs‐CRP/CRP concentrations were similar in patients with MINOCA compared with MI‐CAD, which might be attributed to lack of adjusted comparisons in these studies or differences in blood sampling approaches during the index hospitalization.

GDF‐15 is a stress‐responsive member of the transforming growth factor‐β cytokine superfamily and is a well‐established marker of poor prognosis and future adverse events in patients with MI but also other cardiovascular disease.^17^, ^18^, ^19^ It is expressed in many tissues in different pathological conditions, although the pathobiology in MI is not fully understood.^19^ GDF‐15 is upregulated in ischemic cardiomyocytes in rat/mouse models and humans and has been associated with inflammation, oxidative stress, and hypoxia.^19^ In correspondence with hs‐CRP, GDF‐15 concentrations were initially higher in patients with MINOCA compared with MI‐CAD. In contrast, however, mean GDF‐15 concentrations consistently decreased after the first 25 hours, indicating the involvement of different pathways in the secretion of these 2 biomarkers. Indeed, GDF‐15 concentrations at the acute MI event seem not to be independently related to the extent of myocardial injury, as reflected by necrosis biomarkers, such as cardiac troponin, or cardiac magnetic resonance imaging.^19^

An interesting observation was that the duration from onset of symptoms to blood sampling was substantially longer for patients with MINOCA than for patients with MI‐CAD. This might depend on a longer delay for patients with MINOCA to seek medical attention or a slower response from caregivers. Such structural biases might also have been present in other studies investigating biomarker concentrations in mixed populations with MI (ie, not only consisting of patients with MINOCA or MI‐CAD but also, and more important, of patients with STEMI and NSTEMI). In the present study, patients with NSTEMI experienced a 2‐fold to 3‐fold longer time to blood sampling from the onset of symptoms. Moreover, bear in mind that patients with STEMI and NSTEMI, in addition to MINOCA and MI‐CAD, displayed rather different biomarker dynamics of hs‐cTnT, NT‐proBNP, and hs‐CRP during the first 50 hours. Accordingly, time from symptom debut to blood sampling, MINOCA/MI‐CAD status, and STEMI/NSTEMI status need to be considered when evaluating biomarker concentrations in patients with MI. Indeed, the importance of considering these 3 variables was further substantiated by the significant global tests of interaction in the spline models.

### Biomarker Concentrations at 1 Month After the Index MI and Long‐Term Changes

The notion of a more favorable pathophysiological response in patients with MINOCA compared with MI‐CAD was supported by 1‐month biomarker concentrations and their changes from the acute phase of MI. More pronounced decreases for hs‐cTnT, NT‐proBNP, and hs‐CRP were noted in patients with MINOCA. At 1‐month follow‐up, concentrations of these biomarkers were lower both numerically and following adjustment. Taken together, this suggests less remaining myocardial injury, dysfunction, and inflammation/remodeling in the stable phase after MINOCA. However, as noticed both in the present study and in previous studies, GDF‐15 concentrations remain fairly constant from the acute to stable phase after acute coronary syndrome,^31^, ^32^ in contrast to hs‐CRP, cardiac troponin, and natriuretic peptides.^31^ Furthermore, the change in GDF‐15 concentrations and levels at 1‐month follow‐up did not differ between patients with MINOCA and MI‐CAD. Consequently, GDF‐15 seems to demonstrate more chronic pathophysiological processes that are less influenced by the acute event and whether the event is MINOCA or MI‐CAD.

Biomarker concentrations in the stable phase after an episode of MINOCA have only been investigated in the SMINC (Stockholm Myocardial Infarction With Normal Coronaries) study,^8^, ^9^ to the best of our knowledge. In the SMINC study, 3‐month B‐type natriuretic peptide^9^ and NT‐proBNP^8^ concentrations were lower in patients with MINOCA compared with MI‐CAD, which is in line with the results of the current study. Also, hs‐CRP concentrations were lower in SMINC study in patients with MINOCA,^9^ despite having a numerically higher prevalence of inflammatory conditions compared with the cohort with MI‐CAD.^8^, ^9^ In contrast, however, inflammatory biomarkers other than hs‐CRP differentiated MINOCA from MI‐CAD, suggesting greater inflammatory activation after MINOCA.^9^ The longer time period for biomarker sampling after the index event in the SMINC study may explain part of this discrepancy. Nonetheless, alternative diagnostic modalities, such as analysis of novel inflammatory biomarkers or imaging methods,^27^, ^28^, ^33^ might be considered when closer evaluating such a complicated process as myocardial inflammation in the post‐MI phase.

### Strengths and Limitations

The present study has several strengths. First, the study population was obtained from the PLATO trial, a large randomized control trial in which patients were carefully included and monitored. Second, we studied an international cohort with MINOCA, because the PLATO trial included patients from 862 centers in 43 countries^21^ from Europe, North, Central, and South America, Asia, and Australasia, strengthening the generalizability of our results. Third, we examined biomarkers that are established in MI and have well‐defined pathophysiological mechanisms, except perhaps for GDF‐15.^17^, ^18^, ^19^ Fourth, available information on symptom onset allowed for analyses of temporal biomarker changes during the acute phase of MINOCA. However, we acknowledge that timing variables may be imprecise estimates of the event of myocardial injury in patients with NSTEMI.

There are also some limitations to our study. First, the study cohort stems from a randomized control trial and, thus, probably represents a healthier subset of the general population with MI.^34^ Second, the diagnoses of the index acute coronary syndrome and MI were determined by the investigators at each PLATO trial site without central adjudication, and patients were retrospectively classified as having MINOCA or MI‐CAD. Accordingly, we lack information on the indication for coronary revascularization procedures performed in 46 patients counted as having MI‐CAD but having coronary stenoses <50% (Figure 1). Possible reasons include incorrectly stated stenosis degree in the case report form, a coronary plaque with ≥50% stenosis that was considered hemodynamically significant by fractional flow reserve, or intercurrent complications, such as iatrogenic coronary dissection or thrombosis. Moreover, data on findings from echocardiography and cardiac magnetic resonance imaging examinations were not available, so we cannot exclude the possibility that some patients with MINOCA experienced Takotsubo syndrome or myocarditis. Also, the extent of potential coronary spasm or microvascular dysfunction, as assessed by invasive coronary testing, is unknown. Third, we only investigated 4 biomarkers and might have gained further insights if other markers of inflammation, myocardial dysfunction, or injury had been added. Furthermore, there are many other pathophysiological processes in MI (ie, thrombocyte and coagulation activation) that were not covered by studying only the current biomarkers. Fourth, the washout effect on biomarker concentrations might have been more prominent in patients with MINOCA because all blood samples were collected before revascularization of patients with MI‐CAD, including patients with occlusion of the culprit artery. This effect might have influenced all biomarker concentrations at baseline, although probably hs‐cTnT in particular, possibly resulting in higher concentrations in patients with MINOCA in proportion to patients with MI‐CAD. Fifth, in the study, we considered the patients with MINOCA as a homogeneous group, whereas it is recognized that MINOCA has different pathophysiological causes.^1^, ^2^, ^5^, ^6^ Sixth, the biomarker dynamics during the first 50 hours from symptom onset were based on one blood sample from different patients in the PLATO trial instead of serial blood sampling of the same patients. Seventh and finally, although we included all available blood samples at 1 month after the index MI, the number of patients studied at follow‐up was small and may have been subject to selection bias as the patients were somewhat healthier than the baseline population. However, differences in clinical characteristics between patients with MI‐CAD and MINOCA were similar at both baseline and follow‐up. Also, adjustment for differences in medications at follow‐up did not alter the 1‐month biomarker results.

## CONCLUSIONS

The concentrations and temporal changes of hs‐cTnT, NT‐proBNP, hs‐CRP, and GDF‐15 at the index MI and 1 month later suggest differences in the pathobiology between MINOCA and MI‐CAD. Our data indicate greater initial inflammatory activity, similar degree of myocardial dysfunction, and less pronounced myocardial damage in patients with MINOCA compared with MI‐CAD but also more transient processes in patients with MINOCA. Further analyses are needed to explore the underlying pathophysiological processes and potential implications on patient management.

## Sources of Funding

The present study was supported by the Swedish Foundation for Strategic Research (RB13‐0197) and Swedish Association of Local Authorities and Regions. The PLATO (Platelet Inhibition and Patient Outcomes) trial and the biomarker analyses were sponsored by AstraZeneca. The authors are solely responsible for the design and conduct of this study, all study analyses, the drafting and editing of the manuscript, and its final contents.

## Disclosures

Dr Giannitsis reports grants and personal fees from Roche Diagnostics and Daiichi Sankyo; personal fees from Bayer Vital, Boehringer Ingelheim, AstraZeneca, BRAHMS Deutschland, Novo Nordisk, Idorsia, Radiometer, and Mitsubishi Chemicals, outside the submitted work. Dr Budaj reports personal fees and nonfinancial support from AstraZeneca, during the conduct of the study; personal fees and nonfinancial support from Bristol Myers Squibb and Sanofi Aventis; personal fees from Eisai, Novartis, GlaxoSmithKline, and Amgen; personal fees and nonfinancial support from Bayer, outside the submitted work. Dr Katus reports personal fees from Bayer, AstraZeneca, and Daiichi, outside the submitted work. Dr Storey reports research grants and personal fees from AstraZeneca, Cytosorbents, GlyCardial Diagnostics, and Thromboserin; personal fees from Alnylam, Bayer, Bristol Myers Squibb/Pfizer, Chiesi, CSL Behring, HengRui, Idorsia, Intas Pharmaceuticals, Medscape, Novartis, PhaseBio, Portola, and Sanofi Aventis. Dr Cornel reports membership of advisory boards with Amgen and AstraZeneca. Dr Siegbahn reports institutional research grants from AstraZeneca, Boehringer Ingelheim, Bristol‐Myers Squibb/Pfizer, GlaxoSmithKline, and Roche Diagnostics, outside the submitted work. Dr Hjort, Dr Eggers, T. Ghukasyan Lakic, J. Lindbäck, Dr Becker, Dr Wallentin, and Dr Lindahl have nothing to declare.

## Supporting information

Data S1
Table S1
Figure S1–S2
