# Supplementary material for: Biomarker Concentrations and Their Temporal Changes in Patients With Myocardial Infarction and Nonobstructive Compared With Obstructive Coronary Arteries: Results From the PLATO Trial
Source: J Am Heart Assoc. 2022 Dec 24;12(1):e027466. doi: 10.1161/JAHA.122.027466 (PMC9973579; doi:10.1161/JAHA.122.027466)
Supplement: Supplementary file 1 — Data S1 Table S1 Figure S1–S2 [file JAH3-12-e027466-s001.pdf]

SUPPLEMENTAL MATERIAL

The PLATO Trial Investigators

PLATO principal investigators by country

Argentina

Pablo Schygiel (Instituto Médico Adrogúe, Adrogué; 6)
Oscar Allall (Hospital Córdoba, Córdoba; 16)
Hugo Ramos (Clínica Privada Caraffa, Córdoba; 12)
Julio Bono (Sanatorio Allende, Córdoba; 9)
Juan Fuselli (CEMIC, Buenos Aires; 4)
Carlos Cuneo (Hospital San Bernardo, Salta; 1)
Hugo Colombo (Clínica Privada Colombo, Córdoba; 6)
José Navarro Estrada (Hospital Italiano, Autonomous City of Buenos Aires; 7)
Carlos Pasinato (Complejo Médico-Policial "Churruca-Visca", Buenos Aires; 3)
Daniel Nul (PBA - I Medico Constituyentes - Cardiology, Morón; 20)
Mario Berli (Hospital Cullen, Santa Fé; 37)
Rodolfo Milesi (Instituto Cardiovascular Santa Fé, Santa Fé; 38)
Miguel Hominal (Sanatorio Médico de Diagnóstico y Tratamiento, Santa Fé; 17)
Ricardo Fernandez (Sanatorio San Gerónimo, Santa Fé; 52)
Adrian Hrabar (Sanatorio Modelo de Quilmes, Quilmes; 13)
Hector Luciardi (Hospital Centro de Salud, Tucuman; 6)
Juan Muntaner (Centro Modelo de Cardiologia, Tucuman; 22)
Gerardo Zapata (Instituto Cardiovascular de Rosario, Rosario; 20)
Stella Macin (Instituto de Cardiología de Corriente "J.F.Cabral", Corrientes; 24)
Marcelo Bettinoti (Sanatorio Güemes, Buenos Aires; 27)
Jorge Julio Bluguermann (Policlinica Bancaria, Autonomous City of Buenos Aires; 16)
Eduardo Gabriel Hasbani (Centro Privado de Cardiología, San Miguel de Tucumán; 21)
Ruben Piraino (Sanatorio Plaza, Rosario; 18)
Fernando Colombo Berra (Sanatorio de la Trinidad Quilmes, Quilmes; 14)
Alejandro Garcia Escudero (Clinica Espora, Adrogue; 1)

Australia

Julian Vaile (Flinders Medical Centre, Bedford Park; 9)
Darren Walters (Prince Charles Hospital, Chermside; 22)
Tony Dart (Alfred Hospital, Melbourne; 4)
Chris Hii (Calvary Health Care ACT, Melbourne; 12)
Paul Garrahy (Princess Alexandra Hospital, Woolloongabba; 21)
John Amerena (Geelong Hospital, Geelong; 4)
John Counsell (Dandenong Hospital, Dandenong; 4)
Leonard Arnolda (Royal Perth Hospital, Perth; 7)

Austria

Gerald Maurer (AKH Wien, Wien; 20)
Kurt Huber (Wilhelminenspital der Stadt Wien, Wien; 63)
Franz Roithinger (Thermenklinikum Mödling, Mödling; 5)
Josef Hofer (LKH Freistadt, Freistadt; 9)
Helmut Brussee (Med.-Univ. Graz, Graz; 9)
Heinz Krappinger (LKH Villach, Villach; 1)
Joachim Nesser (KH Elisabethinen Linz, Linz; 3)
Peter Siostrzonek (KH der Barmherzigen Schwestern Linz, Linz; 8)
Otmar Pachinger (Med.-Univ. Innsbruck, Innsbruck; 19)
Heinz Drexel (LKH Feldkirch, Feldkirch; 7)

Belgium

Frank Cools (A.Z. Klina, Brasschaat; 15)
Michel Eycken (AZ Sint Augustinus, Wilrijk; 2)

Marcelo Goldstein (Clinique Sainte-Anne Saint-Remi, Brussels; 14)
Patrizio Lancellotti (Centre Hospitalier Universitaire Sart Tilman, Liège; 11)
Frédéric Mathieu, Pierre Materne (Centre Hospitalier Régional de la Citadelle, Liège; 15)
Erwin Raymenants (AZ Sint-Maarten, Mechelen; 10)
Walter Van Mieghem (Ziekenhuis-Oost Limburg Campus Sint-Jan, Genk; 11)
Mark Claeys (Universitair Ziekenhuis Antwerpen, Edegem; 11)
Edouard Benit (Virga Jesse Ziekenhuis, Hasselt; 3)
Philippe Dubois (Centre Hospitalier Universitaire de charleroi, Charleroi; 5)
Dia El Allaf (Centre Hospitalier Hutois, Nuy; 36)
Guy Heynickx (O.L.V. Ziekenhuis, Aalst; 15)
Harry Striekwold (Heilig Hartziekenhuis Mol, Mol; 18)
Patrick Timmermans (Clinique Saint-Luc, Bouge; 4)

<u>Brazil</u>

Felipe Lima (HC FMUSP Incor, São Paulo; 63)
Leopoldo Piegas (Instituto Dante Pazzanese, São Paulo; 6)
Paulo Lotufo Hospital (Universitário da Universidade de São Paulo, São Paulo; 17)
Valdir Golin (Hospital Irmandade Santa Casa de Misericordia de Sao Paulo, São Paulo; 8)
Antônio Carvalho (Universidade Federal de São Paulo, São Paulo; 11)
Lilia Maia (Fundacao da Faculdade Regional de Sao Jose do Rio Preto, São José do Rio Preto; 14)
Gilmar Greque (Instituto de Moléstias Cardiovasculares, São José do Rio Preto; 6)
José Francisco Saraiva (Hospital e Maternidade Celso Pierro, Campinas; 16)
Peo Filho (Hospital Conceição, Porto Alegre; 29)
Oscar Dutra (Instituto de Cardiologia do Rio Grande do Sul, Porto Alegre; 54)
Jorge Ribeiro (Hospital de Clinicas de Porto Alegre, Porto Alegre; 4)
Paulo Leaes Irmandade Santa Casa de Misericórdia de Porto Alegre, Porto Alegre; 28)
Euler Manenti (Hospital Mãe de Deus, Porto Alegre; 4)
Jose Antonio Abrantes (Santa Casa de Misericórdia de Pelotas, Porto Alegre; 34)
Paulo Roberto Rossi (Hospital Universitário Evangélico de Curitiba, Curbita; 18)
Harry Filho (Inst. Cardiologia de Santa Catarina, São José; 10)
Denilson Albuquerque (Fundacao Cardio Vascular Pedro Ernesto, Rio de Janeiro; 6)
Gilmar Reis (Irmandade Santa Casa de Misericordia de Belo Horizonte, Belo Horizonte; 60)
Álvaro Rabelo (Fundação Baiana de Cardiologia, Salvador; 16)
Marcelo Teixeira (Hospital Português, Salvador; 2)
Délcio JúniorHospital Universitário da Univ. Federal de Mato Grosso, Campo Grande; 33)
Maria Fernanda Garcia (Instituto do Coração do Distrito Federal, Brasília; 4)
Ludmilla Almeida Oliveira (Pro Matre - Instituto do Coração (InCor), Natal; 11)
Maria Sanali Paiva (Natal Hospital Center, Natal; 11)
Yorghos Michalaros (Instituto Previdência Servidores de Minas Gerais, Belo Horizonte; 40)
João Batista Júnior (Hospital Agamenon Magalhães, Recife; 31)
Raul Júnior (Hospital Santa Rita, Maringá; 32)
Hans Fernando Dohmann (Hospital Pró-Cardíaco (PROCEP), Rio de Janeiro; 3)
Salvador Rassi (Hospital das Clínicas da Universidade Federal de Goiás, Goiânia; 18)
Antonio Carlos Sousa (Hospital São Lucas, Aracaju; 23)
Marco Gomes (Hospital Memorial Arthur Ramos, Maceió; 22)
Eduardo Maia (Sociedade Espanhola de Beneficencia, Rio de Janeiro; 2)
José Neto (Irmandade Santa Casa de Misericórdia de Curitiba (PUC-PR), Curitiba; 16)
Roberto Botelho (Hospital Santa Catarina, Uberlândia; 41)

<u>Bulgaria</u>

Dimitar Raev (Central Clinical Hospital, Sofia; 30)
Assen Goudev (MHAT Queen Joanna, Sofia; 24)
Sotir Marchev (5th MHAT, Sofia; 22)
Atanas Mihov (MHAT St. Ekaterina, Dimitrovgrad; 20)
Anastas Popov (MHAT Haskovo, Haskovo; 13)
Maria Cekova (MHAT "Georgi Stranski" Cardiology Dept and ICU, Pleven; 52)
Silvia Dimitrova (MHAT Russe, Ruse; 13)

Stefan Denchev (MHAT Alexandrovska, Sofia; 30)
Mladen Grigorov (2nd MHAT, Sofia; 23)
Snezhanka Tisheva (MHAT "Georgi Stranski" Cardiology and Rheumatology, Pleven; 55)
Atanas Penev (MHAT 'St. Marina', Varna; 13)
Dobri Hazarbasanov (MHAT 'St. Anna', Sofia; 16)
Stanislav Petranov (MHAT Burgas, Burgas; 20)
Stanislav Petranov (MHAT Plovdiv , Plovdiv; 11)
Boicho Boichev (MHAT Kazanlak, Kazanlak; 25)
Nina Shehova (MHAT Dr.Bratan Shukerov, Smolian; 35)
Varbitza Hergeldjieva (MHAT Russe, Russe; 10)
Toma Vladimirov (MHAT Gabrovo, Gabrovo; 2)
Fedya Nikolov, Atanas Djurdjev (UMHAT St.Georgi, Plovdiv; 37)

Canada

Pierre Theroux (Institut de Cardiologie de Montreal, Montréal; 12)
James Swan (Scarborough Cardiology Research, Scarborough; 64)
Barry Rose (The Health Science Centre, St John´s; 9)
Christian Constance (Hopital Maisonneuve-Rosemont, Montreal; 27)
Krishnan Ramanathan (Diamond Health Care Centre, Vancouver; 6)
Wolf Peter Klinke (Victoria Heart Institute, Victoria; 18)
Warren Cantor (Southlake Regional Health Centre, Newmarket; 21)
Robert Welsh (Edmonton University Hospital, Edmonton; 9)
John Ducas (St Boniface General Hospital, Winnipeg; 1)
Petr Polasek (Kelowna Cardiology Research, Kelowna; 2)
Thao Huynh Thanh (Hopital General de Montreal, Montreal; 5)
Rakesh Bhargava (Lakeridge Health Oshawa, Oshava; 16)
Robert Teskey (N.B. Heart Centre, Saint John; 20)
Saul Vizel (Cambridge Memorial Hospital, Cambridge; 23)
John Paul Deyoung (Medical Centre Cornwall, Cornwall; 19)
Hubert Comtois (Cite de la Sante de Laval, Laval; 18)
Patrick Ma (Rockyview Hospital, Calgary; 5)
Robert Dupuis (Centre Hospitalier de la Region de I Amiante, Thetford mines; 10)
John Heath (Campbell River Hospital, Campbell River; 2)
Danielle Dion (Centre Hospitalier Beauce-Etchemin, Saint-Georges; 4)
David Cleveland (Penticton Regional Hospital, Penticton; 3)
Dennis Rupka (Fraser Clinical Trials, New Westminster; 10)
Shyam Radhakrishnan (Sunnybrook Health Sciences Centre, Toronto; 9)
Jayant Bhatt Brockville (General Hospital, Brockville; 28)
Rodney Zimmermann (Regina General Hospital, Regina; 13)
Michel Nguyen (C.U.S.E. Site Fleurimont, Sherbrooke; 2)
Josep Rodes Cabau (Hopital Laval, Quebec City; 22)
Michel Le May (Ottawa Heart Institute, Ottawa; 8)
William Kostuk (London Health Sciences Centre, London; 12)
Jan Kornder (Surrey Memorial Hospital, Surrey; 3)

China

Jilin Chen (Fu Wai Hospital, Beijing; 11)
Xinchun Yang (Beijing Chaoyang Hospital, Beijing; 61)
Shuzheng Lv (Anzhen Hospital, Beijing; 22)
Yong Huo (Peking University First Hospital, Beijing; 28)
Jianhua Zhu (Hospital of Zhejiang, Hang Zhou; 19)
Zhanquan Li (The Peoples Hospital of Liaoning Province, Shenyang; 66)
Dejia Huang (Chengdu Hua Xi Hospital, Chengdu; 50)
Jiyan Chen (Guang Dong Provincial Peoples Hospital, Guangzhou; 41)
Ben He (Renji Hospital, Shanghai; 42)

Qi Hua (Xuanwu Hospital, Beijing; 65)
Yaling Han (Shenyang SY P.L.A General Hospital, Shenyang; 13)

Czech Republic

Jindrich Spinar (Fakultni nemocnice Brno-Bohunice, Brno; 93)
Milos Holub (Nemocnice Jihlava, Jihlava; 8)
Eva Mandysova (Nemocnice Na Homolce, Prague; 11)
Jan Belohlavek (Vseobecna fakultni nemocnice, Prague; 120)
Petr Reichert (Nemocnice Teplice, Teplice; 57)
Ondrej Cermak (Nemocnice Slany, Slany; 55)
Vratislav Dedek (Nemocnice Usti nad Orlici, Usti nad Orlici; 33)
Pavel Cervinka (Masarykova nemocnice, Usti nad Labern; 50)
David Horak, Frantisek Holm (Krajska nemocnice Liberec, Liberec; 161)
Ladislav Groch (Fakultni nemocnice u Sv. Anny, Brno; 87)
Zuzana Motovska (Fakultni nemocnice Kralovske Vinohrady, Prague; 145)
Rudolf Spacek (Nemocnice na Frantisku, Prague; 4)
Jan Matejka Nemocnice (Pardubice, Pardubice; 45)
Leos Pleva, Roman Stipal (Ostrava-Poruba - FN, Ostrava-Poruba; 14)
Marek Richter (Fakultni nemocnice Olomouc, Olomouc; 17)
Jiri Povolny (Nemocnice Kladno, Kladno; 7)
Jan Vojacek (Fakultni nemocnice Hradec Kralove, Hradec Kralove; 20)
Jiri Vejvoda (FN Motol, Kardiovaskulární centrum FN Motol, Prague; 55)
Zdenek Coufal (Zlin - Krajska nem. T.Bati, a.s, Zlin; 12)
Zdenek Monhart (Nemocnice Znojmo, Znojmo; 27)

Denmark

Per Dahl Christensen (Viborg-Kjellerup Sygehus, Viborg; 6)
Else Sørensen, Karen Dodt (Horsens Sygehus, Horsen; 5)
Lars Frost (Regionshospitalet Silkeborg, Silkeborg; 17)
Dorthe Dalsgaard (Regionshospitalet Herning, Herning; 12)
John Markenvard (Fredericia Sygehus, Fredericia; 5)
Ole Nyvad (Fredericia og Kolding Sygehuse, Kolding; 1)
Knud Pedersen (Odense Universitetshospital, Odense; 8)
Roman Sykulski (Storstrømmens sygehus, Næstved; 26)
Kim Klarlund (Køge Sygehus, Køge; 35)
Carl Dahlstrøm (Sygehus Syd, Slagelse; 14)
Thomas Melchior (Roskilde Sygehus, Roskilde; 8)
Tomas Jakobsen (Hillerød Sygehus, Hillerød; 10)
Jan Jensen (Gentofte Amtssygehus, Hellerup; 39)
Knud Skagen (Herlev Hospital, Herlev; 10)
Søren Rasmussen (Hvidovre Hospital, Hvidorvre; 9)
Jens Erik Nielsen (Glostrup Hospital, Glostrup; 32)
Jens Brønnum-Schou (Amager Hospital, Copenhagen; 26)
Peer Grande (Rigshospitalet, Copenhagen; 55)
Christian Tuxen (Frederiksberg Hospital, Frederiksberg; 12)
Tonny Nielsen (Sydvestjysk sygehus, Esbjerg; 26)
Jens Petersen (Sygehus Vendsyssel, Hjørring; 8)
Natasha Roseva-Nielsen (Sygehus Vestsjælland Holbæk, Holbæk; 13)
Allan Mcnair, Margrethe Ege Olsen (Frederikssund Sygehus, Frederikssund; 3)
Henrik Nielsen (Bispebjerg Hospital, Copenhagen; 2)

Finland

Ilkka Tierala (HUS, Meilahden sairaala, Helsinki; 6)
Antti Ylitalo (Satakunnan keskussairaala, Pori; 10)

Seppo Utriainen (Etelä-Karjalan keskussairaala, Lappeenranta; 2)
Juha Hartikainen (Kuopin yliopistollinen sairaalam, Kuopio; 15)
Juhani Airaksinen (Turun Yliopistollinen Keskussairaala, Turku; 30)
Seppo Voutilainen (Päijät-Hämeen keskussairaala, Lahti; 14)
Kai Nyman (Keski-Suomen keskussairaala, Jyväskylä; 44)
Mikko Halkosaari (Keski-Pohjanmaan keskussairaala, Kokkola; 9)
Alexandre Hadjikov (Lapin keskussairaala, Rovaniemi; 24)

France

Jean-Pierre Bassand (Hopital Jean Minjoz, Besancon; 18)
Jean-Ernst Poulard (Centre Hospitalier Général dAbbeville, Abbeville; 10)
Nourredine El Mansour (Nouvel Hôpital - Usic, Valenciennes; 73)
Fabrice Leroy (Centre Hospitalier de Douai, Douai; 11)
Gilles Traisnel (Polyclinique du Bois, Lille; 13)
Eric Decoulx (Hopital de Tourcoing Guy Chatiliez, Tourcoing; 20)
Damien Maes (Clinique du Parc, Croix ; 2)
Xavier Lamit (Hôpital Intercommunal de Fréjus Saint Raphaël, Fréjus; 2)
Eric Maupas (Hôpital Privé les Franciscaines, Nîmes; 4)
Gilles Bayet (Clinique Rhone Durance, Avignon; 10)
Karim Gacem (H Marengo, Chloet; 11)
Claude Cassat (CHU Dupuytren, Limoges; 4)
Pierre Coste (Hôpital Cardiologique du Haut Lévêque, Pessaq; 7)
Nicolas Delarche (Centre Hospitalier François Mitterrand, Pau; 11)
Laurent Ledain (Centre Hospitalier St Louis, La Rochelle; 3)
Gilles Mougeot (CHG de Senlis, Senlis; 13)
Gery Hannebicque (Centre Hospitalier d´Arras, Arras; 17)
Riadh Rihani (Centre Hospitalier Saint Philibert, Lomme; 13)
François Philippe (Institut Mutualiste Montsouris, Paris; 4)
Jean-Jacques Blanc (Hopital de la cavale Blanche, Brest; 15)
Bruno Farah (Clinique Pasteur, Toulouse; 17)
Hervé Le Breton (Che Pontchaillou, Rennes; 3)
René Koning (Clinique Saint Hilaire, Rouen; 5)
Patrick Henry (Hopital Lariboisiere, Paris; 7)
Michèle Escande (CH Allauch, Allauch; 5)
Jean-Luc Dubois-Rande (Hôpital Henri Mondor, Creteil; 7)
Simon Elhadad (Centre Hospitalier de Lagny sur Marne, Lagny sur Marne; 48)
Gilles Grollier (CHU Côte de Nacre, Caen; 9)
Yves Cottin (CHU Bocage, Dijon; 15)
Philippe Garot (Hôpital Claude Galien, Quincy sous Sénart; 10)
Olivier Dibon (Hôpital de la Source, Orleans; 16)
Jean-François Rousseau (CHG Bercagnes, Falaise; 3)
Jean Noel Labeque (Centre Hospitalier Cote Basque, Bayonne; 3)
Patrick Ohlmann (Hôpital de Hautepierre, Strasbourg; 7)
Patrick Khanoyan (Hôpital Saint Joseph, Marseille; 8)

Georgia

Vakhtang Chumburidze (National Center of Therapy, Tblisi; 35)
Kakhi Paposhvili (Clinical Hospital 2, Tblisi; 42)
Irakli Megreladze (Cardiology Clinic, Tblisi; 70)
Bondo Kobulia (Institute of Cardiology, Tblisi; 17)
Nodar Emukhvari (Clinic 1, Tblisi; 39)
Anzor Melia (Cardiological Clinic "GULI", Tblisi; 25)
Merab Mamatsashvili (Clinic "ADAPTI", Tblisi; 52)
Gulnara Chapidze (Emergency Cardiology Centre, Tblisi; 101)
George Khabeishvili (Diagnostic Services Clinic, Tblisi; 138)

Germany

Evangelos Giannitsis (Universitätsklinikum Heidelberg, Heidelberg; 50)
Harald Darius (Vivantes Netzwerk für Gesundheit GmbH, Berlin; 4)
Jürgen Dahl (Kliniken Mariahilf GmbH, Mönchengladbach; 39)
Norbert Franz (Schüchtermann Klinik, Bad Rothenfelde; 17)
Ruth H Strasser (Universitätsklinik an der TU Dresden, Dresden; 6)
Mathias Borst (Caritas-Krankenhaus Bad Mergentheim GmbH, Bad Mergentheim; 9)
Karl-Heinz Kuck (Proresearch Klinische Forschung und Entwicklung, Hamburg; 6)
Hartmut Gülker (Helios Klinikum Wuppertal, Wuppertal; 281)
Stephan Lüders (St. Josefs-Hospital Cloppenburg, Cloppenburg; 4)
Sabine Genth-Zotz (Klinikum der Johannes - Gutenberg - Universität, Mainz; 33)
Hubertus Heuer (St Johannes Hospital, Dortmund; 158)
Michael Buerke (Martin-Luther-Universität Halle-Wittenberg, Halle; 7)
Andreas Jeron, Christian Hengstenberg (Universitätsklinikum Regensburg, Regensburg; 14)
Stephan Felix (Klinikum der Ernst-Moritz-Arndt-Universität Greifswald, Greifswald; 16)
Christoph Nienaber (Universitätsklinikum Rostock, Rostock; 9)
Stavros Konstantinides (Universitätsklinikum der Georg August Universität, Göttingen; 28)
Wolfgang Schöls (Herzzentrum Duisburg, Duisburg; 32)
Markus Lins, Rüdiger Simon (Universitätsklinikum Schleswig-Holstein, Kiel; 11)
Heribert Schunkert (Universitätsklinikum Schleswig-Holstein, Lübeck; 36)
Johannes Brachmann (Klinikum Coburg, Coburg; 35)
Thomas Dorsel (Josephs-Hospital, Warendorf; 19)
Feraydoon Niroomand (Evangelisches Krankenhaus, Mühlheim an der Ruhr; 32)
Thomas Horacek (Forschungszentrum Ruhr/ KliFoCenter GmbH, Witten; 28)
Jörg Kreuzer (St. Vincenz Krankenhaus, Limburg; 9)
Gerian Grönefeld (Asklepios Klinik Barmbek, Hamburg; 2)
Matthias Leschke (Städtische Kliniken Esslingen, Esslingen; 30)
Gudrun Dannberg (Universitätsklinikum Friedrich-Schiller-Universität Jena; 33)
Aydan Yazar Berufsgenossenschaftliche Kliniken Bergmannsheil, Bochum 6
Veselin Mitrovic (Kerckhoff-Klinik Forschungsgesellschaft GmbH, Bad Nauheim; 3)
Jürgen Stumpf (Klinikum Weißer Hirsch, Dresden; 12)
Wolfgang Koenig (Universitätsklinikum Ulm, Ulm; 16)
Roland Prondzinsky (Carl-von-Basedow-Klinikum Merseburg, Merseburg; 4)
Verena Stangl (Charité - Universitätsklinikum Berlin, Berlin; 9)
Ursula Rauch (Charité - Universitätsklinikum Berlin, Berlin; 16)
Paulus Kirchhof (Universitätsklinikum Münster, Münster; 8)
Sven Waßmann, Nikos Werner (Universitätsklinik der Rhein, Bonn; 5)
Christoph Kadel (Städtische Kliniken Frankfurt am Main-Hoechst, Frankfurt; 34)
Rainer Uebis (Klinikum Aschaffenburg, Aschaffenburg; 2)
Jan Monti (Helios Kliniken Berlin - Buch, Berlin; 3)
Oliver Gastmann, Harald Lapp (Helios Klinikum Erfurt GmbH, Erfurt; 29)
Heinrich-Gerhard Klues (Helios Klinikum Krefeld GmbH, Krefeld; 8)
Anette Bühler, Dirk-Ulrich Schaaf (Evangelisches Krankenhaus Königin Elisabeth Herzberge GmbH, Berlin; 5)
Dieter Fischer (Medizinische Hochschule Hannover, Hannover; 1)
Bernhard Grosch (Elisabeth-Krankenhaus, Essen; 1)
Andreas Schäfer (Klinikum der Julius-Maximilians-Universität Würzburg, Würzburg; 9)
Hans-Friedrich Vöhringer (DRK Kliniken Berlin-Köpenick, Berlin; 23)
Martin Dißmann (Vivantes Humbolt-Klinikum, Berlin; 18)

Greece

Dimitrios Kremastinos (Attikon University General Hospital, Athens; 14)
Stefanos Foussas (Tzaneio, General Hospital of Piraeus, Piraeus; 3)
Antonis Manolis (Evangelismos General Hospital of Athens, Athens; 19)

Panagiotis Vardas (University Hospital of Heraklion, Heraklion; 5)
Georgios Theodorakis, Vassilis Voudris (Onassis Cardiac Surgery Center, Athens; 8)
Filippos Triposkiadis (District University General Hospital of Larissa, Larissa; 7)
Dimitrios Alexopoulos (University Hospital of Patra, Patra; 13)
Georgios Parharidis (AHEPA Hospital of Thessaloniki, Thessaloniki; 21)

Hong Kong

Chen Man Ju (Prince of Wales Hospital, Shatin, Hongkong; 16)

Hungary

Anna Czigány (Fovárosi Önkormányzat Nyíro Gyula Kórháza, Budapest; 1)
István Édes (DEOEC Kardio Klinika, Debrecen; 167)
Iván Horváth (PTE Szívgyógyászati Klinika, Pécs; 228)
András Jánosi (Szt. János III. Bel, Budapest; 22)
Ákos Kalina (Állami Egészségügyi Központ Kardiológiai Szakrendelő, Budapest; 11)
András Katona (Pándy Kálmán Megyei Kórház, Gyula; 14)
Zsolt Piróth (Kardiológiai Int., Budapest; 15)
Csaba Király (Kecskemét, Kh. I. Bel, Kecskemét; 127)
Géza Lupkovics (Zala County Hospital Cardiology, Zalaegerszeg; 119)
Mátyás Sereg (Szfvár, Kh. II.Bel, Székesfehérvár; 151)
Béla Oze, László Kássa (Cegléd, Kh. II. Bel, Cegléd; 15)
János Takács (Mosonmagyaróvár, Kh., Mosonmagyaróvár; 7)
Béla Merkely (Semmelweis Egyetem; Ér- és Szívsebészeti Klinika, Budapest; 226)
Ferenc Mágel (Kaposi Mór Oktató Kórház, Belgyógyászat; 3)
Attila Pálinkás (Erzsébet Kórház, Hódmezövásárhely; 9)
András Vértes (Szent István Kórház, II. Belgyógyászat, Budapest; 152)

India:

Prem Pais (St. Johns Medical College Hospital, Bangalore; 9)
Ramesh Babu Byrapaneni (Medwin Hospital, Hyderabad; 10)
Ramesh Babu Pothineni (Citi Cardiac Research Center Ltd, Vijayawada; 7)
Darshan Banker (Bankers Heart Institute, Vadodara; 79)
Praveen Chandra (Max Heart & Vascular Hospital, New Delhi; 9)
Prakash Chandwani (Tongia Heart & General Hospital, Jaipur; 1)
Haridas Kottaram (Amrita Institute of Medical Sciences, Kochi; 8)
Rajendra Kumar Premchand (Krishna Institute of Medical Sciences, Hyderabad; 26)
Sharad Jain (Apollo Hospitals International Ltd., Gandhinagar; 10)
Harshawardhan Mardikar (Spandan Heart Institute and Research Centre, Nagpur; 58)
Padinhare Mohanan (West Fort Hi-Tech Hospital, Thrissur; 13)
Arumugam Chandrakasu, Pradeep Nayar (Frontier Life Line Pvt. Ltd, Chennai; 4)
Ashok Omar (Escorts Heart Institute & Research Center, New Dehli; 17)
Raja Panwar (S.P. Medical College & Hospital, Bikaner; 70)
Keyur Parikh (SAL Hospital & Medical Institute, Ahmedabad; 97)
Swapan Kumar Paul (Peerless Hospital & B.K. Roy Research Center, Kolkata; 2)
Robin Pinto (Holy Family Hospital, Mumbai; 11)
Ramesh Srinivasaiah Saligrama (Bhagwan Mahaveer Jain Heart Centre, Bangalore; 20)
Jitendra Pal Singh Sawhney (Sir Gangarams Hospital, New Dehli; 15)
Skand Trivedi (Bhopal Memorial Hospital & Research Center, Bhopal; 68)

Sudhir Varma (Sadbhavna Medical & Heart Institute, Patiala; 21)
Subramaniam Bhuvaneswaran (PSG Hospitals, Coimabatore; 20)

Indonesia

Anwar Santoso (Sanglah Hospital, Denpasar; 18)
Sunarya Soerianata (National Cardiovascular Center Harapan Kita Hospital, Jakarta; 12)
Erwinanto Erwinanto (Hasan Sadikin Hospital, Bandung; 7)
Sodiqur Rifqi (Telogorejo Hospital, Semarang; 8)
I Gde Suryawan (Soetomo Hospital, Surabaya; 17)

Israel

Ariel Finkelstein (Tel Aviv Sourasky Medical Center, Tel Aviv; 171)
Oscar Kracoff (Kaplan Medical Center, Rehovot; 27)
Shmuel Gottlieb (Bikkur Cholim Hospital, Jerusalem; 23)
Yonatan Hasin (Baruch Padeh Medical Center, Poriya; 133)
Doron Zahger (Soroka Medical Center, Beer Sheva; 20)
Basil Lewis (Lady Davis Carmel Medical Center, Haifa; 13)
Chaim Lotan (Hadassah Ein Kerem Medical Center, Jerusalem; 56)
Lev Bloch(Haemek Medical Center, Afula; 7)
Uri Rosenschein (Bnai Zion Medical Center, Haifa; 34)
Yoseph Rozenman (Wolfson Medical Center, Holon; 2)
Simcha Meisel (Heart institute Hillel Yafe Medical Center, Hadera; 36)
Marc Klutstein (Shaarei Zedek Medical Center, Jerusalem; 99)
Amos Katz (Barzilai medical center, Ashkelon; 17)
Zvi Vered (Assaf Harofeh Medical Center, Zerifin; 3)

Italy

Giampiero Perna (Ospedali Riunti Umberto I - Lancisi- Salesi, Ancona; 7)
Italo De Luca (Azienda Ospedaliera " Ospedale Policlinico Consorziale", Bari; 20)
Antonello Gavazzi (Ospedali Riuniti di Bergamo, Bergamo; 13)
Corrado Tamburino (Azienda Ospedalieria Universitario Vittorio Emanuele - Ferrarotto - Santo Bambino; Catania; 8)
Roberto Ferrari (Arcispedale S. Anna , Ferrara; 6)
Alfredo Zuppiroli (Ospedala Santa Maria Annunziata, Bagno a Ripoli; 9)
David Antoniucci (Azienda Ospedaliera Universitaria Careggi, Careggi; 1)
Di Biase (Ospedali Riuniti di Foggia, Foggia; 2)
Stefano De Servi (Ospedale Civile di Legnano, Legnano; 38)
Roberto Zanini (Azienda Ospedaliera C. Poma, Mantova ; 15)
Antonio Raviele (Ospedale Umberto l, Mestre; 1)
Patrizia Presbitero (Istituto Clinico "Humanitas", Rozzano; 34)
Silvio Klugmann (Azienda Ospedaliera Ospedale Niguarda ca Granda, Milano; 29)
Mariagrazia Modena (Poloclinico di Modena, Modena; 9)
Caso Pio (Azienda Ospedaliera Monaldi, Napoli; 7)
Angelo Sante Bongo (Ospedale Maggiore della Carita, Novara; 11)
Luigi Vignali Azienda (Ospedaliera di Parma, Parma ; 31)
Ezio Bramucci (Ospedale Policlinico S. Matteo, Pavia; 125)
Leonardo Paloscia (Ospedale Civile dello Spirito Santo, Pescara; 64)
Corrado Vassanelli (Ospedale Civile Maggiore, Verona ; 5)
Alessandro Boccanelli (S. Giovanni Addolorata, Roma; 9)
Ezio Giovannini (San Camillo Forlanini, Roma; 3)
Massimo Volpe (Ospedale S. Andrea, Roma; 10)
Raffaele Fanelli (Ospedale Casa Sollievo della Sofferenza, San Giovanni Rotondo; 6)
Pierfranco Terrosu (Ospedale SS Annunziata, Sassari; 44)
Zoran Olivari (Ospedale ca Foncello, Treviso; 26)
Jorge Salerno Uriarte (Azienda Ospedaliera "Ospedale di Circolo e Fondazione Macchi", Varese; 17)
Francesco Fedele (Azienda Universitaria Policlinico Umberto I, Roma; 1)
Franco Mascia (Azienda Ospedaliera S. Sebastiano, Caserta; 46)

Carla Auguadro, Giuseppe Specchia (Policlinico di Monza, Monza; 6)
Fabrizio Ammirati (Ospedale g.b. Grassi, Ostia Lido; 4)
Sergio Berti (Ospedale Pediatrico Apuano G. Pasquinucci, Massa Carrara; 20)

<u>Malaysia</u>

Robaayah Zambahari (Institute Jantung Negara, Kuala Lumpur; 11)
Kui Sim (Sarawak General Hospital, Kuching; 25)
Omar Ismail (Penang General Hospital, Penang; 12)
Wan Azman Wan Ahmad (University Malaya Medical Centre, Kuala Lumpur; 10)

<u>Mexico</u>

Armando García (MTY, IMSS34, Monterrey; 29)
Mario Benavides (Hospital Universitario Eleuterio Gómez, Monterrey; 10)
Gabriel Ramos (Hospital Civil De Guadalajara Dr Juan I Menchaca, Guadalajara; 49)
Ernesto Cardona (Hospital Jardines de Guadalupe, Zapopan; 1)
Pedro Gutierrez Fajardo (Hospital Bernardette, Guadalajara; 1)
Alberto Romo (Centro Médico Nacional de Occidente, Guadalajara; 10)
Carlos Martinez (DF, Cardio, CV, Tlalpan; 18)
Alejandra Meaney (Hospital 1 de Octubre, ISSSTE, Mexico City; 1)
Alejandra Meaney (Hospital Regional Dr Valentin Gomez Farias, ISSSTE, Zapopa; 1)
Raul Velasco (GDL, Angel L., GI, Guadalajara; 2)
Juan Carlos Nuñez (Hospital General de Durango, Durango; 9)
Juan Perez (Hospital General de Puebla SSA, Puebla; 6)

<u>Netherlands</u>

Jan Hein Cornel (Medisch Centrum Alkmaar, Alkmaar; 86)
Freek Verheugt (Universitair Medisch Centrum St. Radboud, Nijmegen; 5)
Marco Alings (Amphia Ziekenhuis, Breda; 27)
Peter Bendermacher (Elkerliek Ziekenhuis, Helmond; 67)
Bryan Van Den Berg (IJsselland Ziekenhuis, Capelle aan den IJssel; 9)
Hugo Beyerbacht (Röpcke-Zweers Ziekenhuis, Hardenberg; 9)
Rolf Michels (Catharina Ziekenhuis, Eindhofen; 6)
René Dijkgraaf (Ziekenhuis Sint Jansdal, Harderwijk; 26)
Marcel Van Der Linde (Ziekenhuis Nij Smellinghe, Drachten; 28)
Reginald Groutars (Sint Lucas Andreas Ziekenhuis, Amsterdam; 18)
Bas Hamer (Meander Medisch Centrum, Amersfoort; 37)
Frank Den Hartog (Ziekenhuis Gelderse Vallei, Ede; 24)
Rob Van Der Heijden (Vlietland Ziekenhuis, Vlaardingen; 20)
Klaas Holwerda (Sint Elisabeth Ziekenhuis, Tilburg; 9)
Maarten Janssen (Kennemer Gasthuis, Haarlem; 11)
Debbie Nicastia, W. Jap-Tjoen-San (Gelre Ziekenhuis, locatie Lukas, Apeldoorn; 2)
Wim Aengevaeren (Rijnstate Ziekenhuis, locatie Arnhem, Arnhem; 34)
Ben Gho (Atrium Heerlen, Heerlen; 22)
Martin De Leeuw (Wilhelmina Ziekenhuis, Assen; 6)
Anno Liem (Oosterschelde Ziekenhuis, Goes; 34)
Hendrik Kruik (Twenteborg Ziekenhuis, Almelo; 26)
Dirk Lok (Deventer Ziekenhuis, Deventer; 53)
Anthonius Oude Ophuis (Canisius Wilhelmina Ziekenhuis, Nijmegen; 63)
René Peters (Tergooi Ziekenhuizen, locatie Blaricum, Blaricum; 11)
Mathijs Pieterse (BovenIJ Ziekenhuis, Amsterdam; 18)
Jacobus Plomp (Ziekenhuis Hilversum, Hilversum; 13)
Jurrien Ten Berg (Sint Antonius Ziekenhuis, Nieuwegei; 13)
Piet Van Rossum (Rivas Medizorg, Gorinchem; 37)
Ramon Robles De Medina (HAGA Ziekenhuis, locatie Leyenburg, den Haag; 16)
Peter Smits (Medisch Centrum Rijnmond-Zuid, Rotterdam; 19)
Attila Dirkali Albert Schweitzer Ziekenhuis, Dordrecht; 11)
Henricus Thijssen (Maxima Medisch Centrum, Veldhoven; 2)

Eric Viergever (Het Groene Hart Ziekenhuis, Gouda; 31)
Adri Withagen (Reinier de Graaf Gasthuis, Delft; 34)
Coen Van Der Zwaan (Ziekenhuis Rivierenland, Tiel; 7)
Ad Van Boven (Medisch Centrum Leeuwarden Zuid, Leeuwarden; 73)
Johannes Waltenberger (Academisch Ziekenhuis Maastricht, Maastricht; 8)

Norway

Leif Erik Erdal, Ole Rolstad (Sykehuset Innlandet HF Lillehammer, Lillehammer; 32)
Ulf Hurtig (Sykehuset Innlandet HF Tynset, Tynset; 5)
Kjell Andersen (Sykehuset Innlandet HF Hamar, Hamar; 18)
Geir Høgalmen (Sykehuset Innlandet HF Gjøvik, Gjøvik; 10)
Bjørn Jørgensen (Asker og Bærum sykehus HF, Rud; 10)
Geir Heggelund (Universitetssykehuset i Nord-Norge HF, Tromsø; 7)
Rune Fanebust (Helse Bergen HF Haukeland universitetssykehus, Bergen; 8)
Gunvald Eivindson (Sørlandet sykehus HF Kristiansand, Kristiansand; 5)
Torstein Gundersen (Sørlandet sykehus HF Arendal, Arendal; 14)
Tor Omland (Sørlandet sykehus HF Flekkefjord, Flekkefjord; 11)
Knut Tore Lappegård (Nordlandssykehuset HF Bodø, Bodø; 4)
Erik Gjertsen (Sykehuset Buskerud HF, Drammen; 11)
Pål Smith (Akershus universitetssykehus HF, Nordbyhagen; 5)
Kjell Berget (Blefjell Sykehus HF Kongsberg, Kongsberg; 9)
Dan Atar (Aker universitetssykehus HF, Oslo; 5)
Nils Lid (Blefjell sykehus Notodden, Notodden; 4)
Lars Gullestad (Rikshospitalet HF, Oslo; 1)

Phillipines

Noe Babilonia (Philippine Heart Center, Quezon City; 28)
John Anonuevo (Philippine General Hospital, Manila; 1)
Richard To (The Medical City, Pasig City; 9)
Gregorio Rogelio (St. Lukes Medical Center, Quezon City; 10)
Edgar Molleno (Perpetual Succour Hospital, Cebu City; 11)
Cesar Estalilla Iii (Cebu Doctors University Hospital, Cebu City; 4)
Raul Martin Coching (Davao Doctors Hospital, Davao City; 15)

Poland

Andrzej Budaj (SPZOZ. Szpital Grochowski im. dr med. Rafała Masztaka, Warszawa; 52)
Pawel Buszman (Wieńcowych SP Szpital, Katowice; 133)
Maciej Dalkowski (Miedziowe Centrum Zdrowia SA, Lubin; 207)
Robert Gil (Centralny Szpital Kliniczny MSWiA, Warszawa; 5)
Andrzej Kleinrok (Szpital Wojewódzki im. Jana Pawła II, Zamosc; 12)
Wlodzimierz Krasowski (Szpital Specjalistyczny św. Wojciecha Adalberta, Gdansk; 34)
Jerzy Lewczuk (Wojewódzki Szpital Specjalistyczny ul, Wroclaw; 53)
Wladyslaw Pluta (Wojewódzkie Centrum Medyczne, Opole; 59)
Roman Szelemej (Specjalistyczy Szpital im.dr Sokołowskiego, Walbrzych; 39)
Hanna Szwed (Klinika Choroby Wieńcowej, Warszawa; 90)
Jacek Gessek (Szpital Miejski im. M. Kopernika, Torun; 81)
Pawel Miekus (Szpital Miejski im. J. Brudzińskiego, Gdynia; 78)
Jacek Kubica (Klinika Kardiologii, Bydgoszcz; 97)
Zygfryd Reszka (Wojewódzki Szpital Zespolony Ul, Elblag; 44)
Malgorzata Krzciuk (Krzciuk Oddział Kardiologii ul, O. Swietokrzyski; 14)
Barbara Kusnierz (Wojewódzki Szpital Specjalistyczny, Bytom; 33)
Marek Piepiorka (Piepiorka Oddział Kardiologii ul., Wejherowo; 44)
Marek Dabrowski (Szpital Bielański ul., Warszawa; 29)
Jerzy Górny (Wojewódzki Szpital Specjalistyczny, Olsztyn; 45)
Piotr Kardaszewicz (Wojewódzk iSzpital Specjalistyczny, Czestochowa; 8)
Jerzy Rekosz (Wojewódzka Stacja Pogotowia, Warszawa; 36)
Waldemar Ruminski (Wojewódzki Szpital Specjalistyczny, Lublin; 4)

Andrzej Rynkiewicz (I Klinika Chorób Serca, Gdansk; 215)
Ryszard Sciborski (Zespół Opieki Zdrowotnej, Olawa; 37)
Pawel Buszman (Polsko-Amerykańskie Kliniki Serca Ul, Bielsko-Biala; 121)
Ryszard Targonski (Internistyczny Miejski Szpital, Olsztyn; 18)
Bozena Wrzosek (Wojewódzki Szpital Specjalistyczny, Radom; 121)
Henryk Wysocki (SP Szpital Kliniczny nr 2, Poznan; 27)
Boguslaw Derlaga (Specjalistyczny Szpital im. E.Szczeklika Ul, Tarnów; 42)
Jan Henryk Goch (Uniwersytecki Szpital Kliniczny nr 3 ul. Sterlinga, Lódz; 3)
Krystyna Jaworska (Wojewódzki Szpital Zespolony im. L. Rydygiera, Torun; 65)
Jerzy Kopaczewski (Szpital Wojewódzki, Wloclawek; 97)
Wladyslaw Sinkiewicz (Wojewódzki Szpital im. dr Biziela ul, Bydgoszcz; 24)
Wieslawa Tracz (Instytut Kardiologii Collegium Medicum UJ Ul, Kraków; 92)
Dariusz Dudek (Szpital Uniwersytecki, Kraków; 49)
Andrzej Drzewiecki (Wojewódzki Szpital Zespolony, Ploc; 90)
Andrzej Hoffmann (Wielospecjalistyczny Szpital Miejski, Bydgoszcz; 29)
Rafal Nizankowski (Katedra Chorób Wewnętrznych Collegium Medicum UJ Ul, Kraków; 18)
Zdzislawa Kornacewicz-Jach (Samodzielny Publiczny Szpital Kliniczny Nr 2 , Szczecin; 50)
Michal Kurowski (SP Wojewódzki Szpital Zespolony, Szczecin; 55)
Walentyna Mazurek (Samodzielny Publiczny Szpital Kliniczny nr 1, Wroclaw; 67)
Wlodzimierz Musial (Szpital Kliniczny Akademii Medyczne, Bialystok; 108)
Marek Bronisz (Oddział Kardiologii PS ZOZ Ul, Inowroclaw; 69)
Michal Szpajer (Szpital Morski im. PCK ul, Gdynia; 74)
Marek Stopinski (Szpital Zachodni im. Jana Pawła II ul, Grodzisk Maz; 2)

Portugal

João Morais (Hospital de Santo André, Leiria; 13)
Isabel Arroja (Centro Hospitalar Lisboa Ocidental-HSF Xavier, Lisboa; 7)
Nunes Diogo (Hospital de Santa Maria, Lisboa; 16)
José Ferreira Santos (Hospital de São Bernardo, Setúbal; 11)
Leitão Marques (Centro Hospitalar Coimbra, Coimbra; 19)
Gonçalo Proença (Hospital Amadora-Sintra, Amadora; 8)
Graça Da Silva (Hospital Distrital de Santarém, Santarém; 7)
Luís Gonçalo (Hospital Padre Américo/Vale do Sousa, Penafiel; 13)
Santos Mendes (Hospital de São Sebastião, Santa Maria da Feira; 4)
Carlos Gonçalves (Centro Hosp Vila Nova Gaia, Vila Nova de Gai; 29)
Pedro Cardoso (Hospital Infante D. Pedro, Aveiro; 17)
Ilídio Moreira (Centro Hospitalar Trás os Montes e Alto Douro, Vila Real; 5)
Luís Miguel Pereira (Hospital S Marcos, Braga; 3)

Romania

Marius Vintila (Sf.Pantelimon, Bucharest; 54)
Mircea Cinteza (UCH Bucharest, Bucharest; 103)
Catalina Arsenescu Georgescu (Centrul de Cardiologie Iasi, Isai; 1)
Radu Capalneanu (Institutul Inimii "Niculae Stancioiu", Cluj Napoca; 2)
Mariana Radoi (Cardiology Brasov, Brasov; 3)
Dan Ionescu (Cardiology Center Craiova, Craiova; 52)
Gabriela Stanciulescu (County Hospital Arges, Pitesti; 110)
Bogdan Minescu (County Hospital Braila, Braila; 34)
Marilena Spiridon (Spitalul Clinic Judetean de Urgenta "Sf.Spiridon", Isai; 20)
Mihai Creteanu (Spitalul Clinic Judetean de Urgenta, Suceava; 11)
Mircea Constantinescu (Spitalul Judetean Buzau, Buzau; 7)

Russia

Alexey Duda (Altay Regional Cardiology Dispensary, Barnaul; 28)
Olga Lesnyak (Sverdlovsk Regional Clinical Hospital 1, Ekaterinburg; 24)
Mikhail Arkhipov (New Hospital Medical Union, Ekaterinburg; 40)
Elena Ovcharenko (Irkutsk Regional Clinical Hospital after Znak Pocheta Order, Irkutsk; 20)
Svatlana Akhunova (Interregional Clinical and Diagnostic Center, Kazan; 20)
Anna Dembitskaya (Kaliningrad Regional Hospital, Kaliningrad; 25)
Olga Barbarash (Kemerovo Cardiology Dispensary, Kemerovo; 36)
Prokhor Pavlov (Khanty-Mansiysk Regional Clinical Hospital, Khanty-Mansiysk; 18)
Sergey Ustyugov, Galina Nechepurenko (Karpovich City Clinical Hospital, Krasnoyarsk; 2)
Kirill Linev (Regional Clinical Hospital, Krasnoyarsk; 3)
Vladimir Shulman (Berzon City Clinical Hospital 20, Krasnoyarsk; 44)
Ivan Gordeev (City Filatov Clinical Hospital 15, Moscow; 36)
Mikhail Ruda (Russian Cardiology Research and Manufacturing Complex, Moscow; 4)
Dmitry Zateyshchikov (Moscow State Healthcare Institution City Hospital 17, Moscow; 28)
Boris Sidorenko (Moscow State Healthcare Institution, City Clinical Hosp 51, Moscow; 2)
Garry Klein (Bayandin Murmansk Regional Clinical Hospital, Murmansk; 29)
Igor Fomin (City Clinical Hospital 5, Nizhniy Novgorod; 12)
Elena Kosmachova (Krasnodar Ochapovsky Regional Clinical Hospital 1, Krasnodar; 13)
Dmitry Belenky (Novosibirsk Clinical Hospital for Emergency Care 2, Novosibirsk; 22)
Vladimir Ganyukov (Novosibirsk Regional Clinical Cardiology Dispensary, Novosibirsk; 15)
Svetlana Baum (Road Clinical Hospital at Novosibirsk-Glavniy Station, Novosibirsk; 11)
Larisa Khaisheva, Ludmila Katelnitskaya (City Hospital for Emergency Care 2, Rostov-na-Donu; 25)
Rumiya Miftyakhova (Samara Regional Clinical Cardiology Dispensary, Samara; 27)
Natalia Burova (Federal Research Center of Heart, Blood and Endocrin, St Petersburg; 32)
Svetlana Boldueva (St.Petersburg Mechnikov State Medical Academy, St Petersburg; 19)
Alexey Sherbak (St.Petersburg City Multiservice Hospital 2, St Petersburg; 6)
Alexander Petrov (Leningrad Regional Clinical Hospital, St Petersburg; 7)
Victor Kostenko (St.Petersburg Dzhanelidze Research Inst for Emergency Care, St Petersburg; 24)
Alexander Filippov (Kirov Military Medical Academy under the Ministry of Defence, St Petersburg; 2)
Alexander Vishnevsky (St.Petersburg City Pokrovskaya Hospital, St Petersburg; 28)
Valentin Markov (Research Cardiology Institute Tomsk Scientific Center, Tomsk; 45)
Vadim Kuznetsov (Tyumen Cardiology Center, Tuymen; 26)
Vladimir Verin (Primorskaya Regional Clinical Hospital 1, Vladivostok; 5)

Singapore

Soo Teik Lim (National Heart Centre, Singapore; 36)
Dinesh Nair, Ing Haan Lim, Jimmy Lim (Tan Tock Seng Hospital, Singapore; 28)

Slovakia

Tibor Duris (Interna klinika FNsP, Nove Zamky; 15)
Viliam Fridrich (Oddelenie intervencnej kardiológie NUSCH, BA, Bratislava; 17)
Pavel Vahala (Interna klinika FN, Nitra; 11)

Pavel Cuncik (Nemocnica A, Wintera, Piestany; 3)
Margita Belicova (Interna klinika, Martinska fakultna nemoc, Martin; 73)
Martin Studencan (Kardiologicke oddelenie VUSCH Kosice, Kosice; 85)
Stanislava Remisova (IV, interna klinika FN a LF, Bratislava; 4)
Peter Kycina (Interne oddelenie NsP, Liptovsky Mikulas; 12)
Vladimir Macek (Interne oddelenie FN, Trnava; 34)
Vladimir Spisak (Interne odd, a odd, arytmii NsP, Zilina; 1)
Miroslav Urban (Interna klinika Ustredna vojenska nemocnica SNP,Ruzomberok; 23)
Marian Hranai (Kardiocentrum s.r.o Nitra, Nitra; 60)

South Africa

Edmund Brice, Hellmuth Weich (Tygerberg Hospital, Cape Town; 38)
Jacobus Badenhorst (Unitas Hospital, Pretoria; 10)
Adrian Horak (Vincent Palloti, Cape Town; 28)
Saleem Dawood (SY Dawood, Cape Town; 27)
Hendrik Theron (Netcare Private Hospital, Bloemfontein; 32)
Tom Mabin (CT, Somerset West, Vergelen, Somerset West; 14)

South Korea

Ki Bae Seung (Kangnam St. Marys Hospital, Seoul; 21)
In Ho Chae, Young-Seok Cho (Seoul National University Bundang Hospital, Seongnam; 3)
Cheol-Whan Lee (Asan Medical Centre, Seoul; 19)
Seung-Jea Tahk (Ajou University Hospital, Suwon; 12)
Jung Han Yoon (Yonsei University Wonju Christian Hospital, Wonju; 20)
Yang-Soo Jang (Yonsei University Severance Hospital, Seoul; 14)
Myung-Ho Jeong (Chonnam University Hospital, Gwangju; 10)
Young Jo Kim (Yeungnam University Medical Center, Daegu; 21)

Spain

Mercé Roque Moreno (H.Clínic i Provincial, Barcelona; 23)
Juan García Picart (H.S.Creu S.Pau, Barcelona; 27)
Eduardo De Teresa Galván (H.Clin.V.Victoria, Málaga; 9)
Carlos Macaya Miguel (H.C.S.Carlos, Madrid; 11)
Andrés Íñiguez Romo (H. de Meixoeiro, Vigo; 6)
Juan Ramón Rey Blas (H.U.La Paz, Madrid; 40)
Miguel Ruano Marco (H. La Fe, Valencia; 25)
Jaume Figueras Bellot (H.Vall d´Hebrón, Barcelona; 26)
Armando Bethencourt González (H. Son Dureta, Palma de Mallorca; 37)
Víctor López García (H. V. Macarena, Sevilla; 14)
Vicente Valentín Segura (H. Dr. Peset, Valencia; 24)
Luis Alberto Batalla Celorio, Pedro Vigil-Escalera González (H.Cabueñes, Gijón; 2)
Fernando Worner Diz (H. Arnau Villanova, Lérida; 32)
Ferrán Jara Clemente (H.Mutua Terrassa, Terrassa; 26)
Gonzalo Marcos Gómez (H.Prov.S.Pedro Alcántara, Cáceres; 15)

Sweden

Stefan James (Akademiska sjukhuset, Uppsala; 46)

Stellan Bandh (Centrallasarettet, Västerås; 59)
Ann Samnegård (Danderyds sjukhus AB, Danderyd; 8)
Pierre Cherfan (Höglandssjukhuset, Eksjö; 35)
Carina Nilsson (Ljungby lasarett, Ljungby; 12)
Jan-Erik Karlsson (Länssjukhuset Ryhov, Jönköping; 18)
Magnus Peterson (Skaraborgs sjukhus, Lidköping; 11)
Thomas Mooe (Östersunds sjukhus, Östersund; 33)
Peter Hårdhammar (Länssjukhuset, Halmstad; 4)
Crister Zedigh (Falu lasarett, Falun; 12)
Mona Lycksell (Länssjukhuset Sundsvall-Härnösand, Sundsvall; 7)
Stefan Rydén (Länssjukhuset, Kalmar; 11)
Magnus Janzon (Universitetssjukhuset, Linköping; 34)
Stefan Jovinge (Universitetssjukhuset, Lund; 11)
Anders Kallryd (Kärnsjukhuset, Skövde; 17)
Carl-Magnus Pripp (Blekingesjukhuset, Karlskrona; 3)
Hans Tygesen (Södra Älvsborgs sjukhus, Borås; 16)
Anders Stjärna (Mälarsjukhuset, Eskilstuna; 11)

Switzerland

Bernhard Meier (Inselspital, Bern; 14)
Tiziano Moccetti (Cardiocentro Ticino, Lugano; 151)
Michael Pieper (Herz- und Neuro-Zentrum Bodensee, Kreuzlingen; 28)
Marco Roffi, Vitali Vérin (Hôpital universitaire Genève, Genève; 4)
Alberto Pagnamenta (Ospedale Regionale di Mendrisio Beata Vergine, Mendrisio; 11)
Augusto Gallino (Ospedale San Giovanni, Bellinzona; 3)
Martin Brack, Hans-Rudolf Baur (Tiefenauspital, Bern; 3)

Taiwan

Ming-Shien Wen (Chang Gung Memorial Hospital - Linkou, Kweishan Shiang; 11)
Wen-Jone Chen (National Taiwan University Hospit, Taipei; 20)
Ying-Hwa Chen (Veterans General Hospital, Taipei; 15)
Charles Hou (Macaky Memorial Hospital, Tapei; 22)
Mien-Cheng Chen (Chang-Gung Memorial Hospital- Kaohsiung, Niao-Song-Shiang; 24)

Thailand

Piyamitr Sritar (Ramathibodi Hospital, Bankok; 25)
Suphot Srimahachota (Chulalongkorn, Bangkok; 24)
Srun Kuanprasert (Maharajnakorn Chiang Mai, Chiang Mai; 55)
Chaiyasit Wongvipaporn (Srinagarind Hospital, Khon Kaen; 30)
Nakarin Sansanayudh (Phramongkutklao, Bankok; 19)

Turkey

Ali Oto (Hacettepe University, Ankara; 2)
Fatih Ertas (Ankara University Medicine Faculty, Ankara; 3)
Ahmet Temizhan (SB Turkiye Yuksek Ihtisas Training and Research Hospital, Ankara; 1)
Zerrin Yigit (Istanbul University, Istanbul; 13)
Mesut Demir (Cukurova University, Adana; 10)

Gokhan Cin (Mersin University, Mersin; 5)
Aytul Belgi (Akdeniz University, Antalya; 5)
Bengi Yaymaci (Kartal Yuksek Ihtisas Education and Research Hospital, Istanbul; 12)

Ukraine

Igor Krayz (Ukrainian Railway Central Clinical Hospital, Kharkov; 58)
Lyudmila Kononenko (Kharkov City Clinical Hospital 27, Kharkov; 20)
Vira Tseluyko (Kharkov City Clinical Hospital 8, Kharkov; 14)
Alexander Parkhomenko (Institute of Cardiology, Emergency and Intensive Therapy, Kiev; 15)
Oleksandr Karpenko (Kiev City Clinical Hospital 1; 16)
Olena Koval Dnepropetrovsk (Emergency Hospital, Dnepropetrovsk; 23)
Leonid Rudenko (Kiev City Clinical Emergency Hospital, Kiev; 17)
Mykola Vatutin (Inst of Urgent and Recovery Surgery DNMU Int Medicine, Donetsk; 4)
Natalyia Kalinkina(Makiyivka City Clinical Hospital No 1 DNMU Int. Dis., Makiyivka; 2)

United Kingdom

Robert Storey (Northern General Hospital, Sheffield; 91)
Greg Lip (City Hospital, Birmingham; 11)
Ranjit More (Victoria Hospital, Blackpool; 46)
Rajdeep Khattar (Manchester Royal Infirmary, Manchester; 15)
Ian Hudson (Glenfield Hospital, Leicester; 3)
Iain Squire (Leicester Royal Infirmary, Leicester; 13)
Mark De Belder (James Cook University Hospital, Middlesborough; 6)
Alastair Pell (Monklands Hospital, Airdrie; 3)
Keith Oldroyd (Western Infirmary, Glasgow; 23)
Chim Lang (Ninewells Hospital, Dundee; 17)
Keith Fox (Edinburgh Royal Infirmary, Edinburgh; 8)
Niall Herity (Belfast City Hospital, Belfast; 13)
Andrew Moriarty (Craigavon Area Hospital, Belfast; 7)
Mark Ramsey (Morriston Hospital, Swansea; 16)
Patrick O'Callaghan (Royal London Hospital, London; 1)
Martin Wilkins (Hammersmith Hospital, London; 4)
Diane Bruce (Poole General Hospital, Poole; 1)
Andrew Bishop (North Hampshire Hospital, Basingstoke; 3)

United States of America

Salvador Lanza (Florida Hospital Altamonte, Orlando; 1)
Theodore Lau (FHS Research Center, Tacoma; 19)
David Henderson, John Walker (Cardiology Research Associates, Ormond Beach; 49)
Michael Imburgia (Louisville Cardiology Medical Group, PSC, Louisville; 21)
Paul Gurbel (Sinai Centre for Thrombosis Research, Baltimore; 10)
Joseph Raffeto (Peninsula Cardiology Associates, Salisbury; 8)
James Donovan (Norton Suburban Hospital Cardiovascular Associates, Louisville; 10)
Venkatesh Nadar (Heritage Cardiology Associates, Camp Hill; 6)
Steven Minor (Austin Heart P.A., San Marcos; 7)
William Rogers (University of Alabama at Birmingham, Birmingham; 2)
Frank Lester (IMC Diagnostic & Medical Clinic Mobile Infirmary, Mobile; 1)
James Bengston (St. Joseph Mercy Oakland Hospital, Pontiac); 8)
Wayne Leimbach (Oklahoma Heart Institute, Tulsa; 7)

Vibhuti Singh (Suncoast Cardiovascular Research, St Petersburg; 10)
Ernesto Rivera (Amarillo Heart Clinical Research Institute, Amarillo; 6)
Ralph Vicari (Holmes Regional Medical Center, Melbourne); 7)
Jerome Anderson (Integris Heart Hospital, Oklahoma City; 2)
Vasilios Papademetriou (VA Medical Center - DC, Washington; 4)
Michael Rich (St Marys Duluth Clinic, Duluth; 8)
Drew Purdy (Black Hills Clinical Research Ctr, Rapid City; 3)
Paul Casale (Lancaster General Hospital, Lancaster; 8)
John Corbelli (Buffalo Cardiology & Pulmonary Associates, Williamsville; 4)
Steven Guidera (Doylestown Hospital, Doylestown; 57)
John Murphy (South County Cardiology Associates Inc, Wakefield; 5)
William Wu (Central Cardiovascular Research Foundation, San Antonio; 4)
John Gordon (San Diego Cardiac Ctr, San Diego; 3)
Biswajit Kar (VA Medical Center - TX, Houston; 16)
Diwakar Lingham, Jorge Davidenko (New York Heart Center, Syracuse; 4)
Brian Friedman (Olathe Medical Center, Olathe; 1)
Michael Lim (St. Louis University Health Sciences Center, St Louis; 16)
Magdi Ghali (Iowa Heart Center PC, West Des Moines; 19)
John Sinden, Marc Silver (Raleigh Cardiology, Raleigh; 3)
Henry Lui (APEX Cardiology PC, Jackson; 22)
Steven Yakubov (Midwest Cardiology Research Foundation, Columbus; 7)
Robert Weiss (Maine Research Associaties, Auburn; 7)
Ajay Virmani (Winchester Cardiology & Internal Medicine Inc., Winchester; 13)
William Matthai (Penn - Presbyterian Medical Center, Philadelphia; 4)
Fayez Shamoon (St. Michael Medical Center, Newark; 2)
Ramin Ebrahimi (VA Greater LA Healthcare System, Los Angeles; 16)
Peter Fattal (Michigan Cardiovascular Institute, Saginaw; 4)
David Kraus (Baptist Clinical Research Center, Memphis; 4)
Radha Sarma (LAC & USC Medical Center, Los Angeles; 2)
Robert Feldman (Munroe Regional Med Center, Ocala; 9)
Mahesh Mulumudi (Providence Everett Med Center, Everett; 1)
Paul Tolerico (Cardiac Diagnostic Associates, York; 6)
John Patterson (Forsyth Medical Center, Winston-Salem; 2)
Wyatt Voyles (Medical Center of the Rockies, Fort Collins; 6)
Kamal Gupta (University of Kansas Medical Center, Kansas City; 1)
Steven Hearne (Delmarva Heart, L.L.C., Salisbury; 10)
David Schneider (University of Vermont, Burlington; 9)
Keith Atassi (Northwest Indiana Cardiovascular Physicians, Valparaiso; 25)
Mahesh Bikkina (St. Josephs Hospital Medical Center, Paterson; 8)
Andrew Doorey (Christiana Hospital, Newar; 4)
Valerian Fernandes (Ralph H Johsnson VAMC, Charleston; 1)
Paul Gordon (Miriam Hospital, Providence; 11)
Gene Langevin (Freeman Health System, Joplin; 24)
Raymond Mckay (Hartford Hospital, Hartford; 18)
Viral Mehta (Comprehensive Cardiovascular Medical Group, Bakersfield; 12)
George Aycock (Cardiology Consultants - FL, Pensacola; 14)
Dominick Angiolillo (University of Florida, Jacksonville; 44)
Barry Reicher (University of Maryland Hospital, Baltimore; 33)
Brian Schwartz (Kettering Medical Center, Kettering; 3)
Annapoorna Kini, Samin Sharma (Mount Sinai Medical Center, New York; 1)
Muhammed Yasin (Integris Southwest Medical Center, Oklahoma City; 16)
Ronald Fields (St. Marys Medical Center, Langhorne; 7)
Jeffrey Popma, Pinak Bipin Shah (Brigham and Womens Hospital - Harvard Medical School, Boston; 4)
Cezar Staniloae (St Vincents Catholic Medical Centers, New York; 4)
Thomas Carlson (Austin Heart P.A., Austin; 9)
Peter Verlee (Northeast Cardiology Associates, Bangor; 10)
Stephen Culp (Sarasota Memorial Hospital, Sarasota; 5)
Massoud Leesar, Sohail Ikram (Univ of Louisville Hospital Jewish Hospital, Louisville; 19)

Saeed Shaikh (Indiana Heart Physicians, Indianapolis; 6)
Bruce Mclellan (St Charles Medical Center Heart Institute of the Cascades, Bend; 11)
Carmelo Panetta, Ganesh Raveenderan (Park Nicollet Heart and Vascular Center, Minneapolis; 2)
Rajesh Dave (Central PA Cardiovascular Research Institute, Harrisburg; 4)
Scott Lieberman (East Texas Medical Center, Tyler; 10)
Abnash Jain, Robert Beto (West Virginia University Ruby Memorial, Morgantown; 1)
Joseph Gelormini (Buffalo Heart Group, Buffalo; 7)
Hildreth Vernon Anderson (Memorial Hermann Hospital, Houston; 2)
Alan Banks (Sentara Norfolk General Hospital, Norfolk; 12)
Harish Chandna (Victoria Heart & Vascular Center, Victoria; 8)
Yellapragada Chandrashekhar (Minneapolis VAMC, Minneapolis; 1)
H Barrett Cheek (Carolina Cardiology Associates, High point; 11)
Michael Del Core (Creighton University, Omaha; 4)
William French (UCLA Medical Center, Torrance; 10)
Jeffrey Harris (McLaren Regional Medical Center, Laper; 1)
David Jinich (Covenant Cardiology Associates, Lubbock; 1)
Kenneth Kent (Suburban Hospital, Bethesda; 17)
Masroor Khan (University of Texas Health Science Center, Tyler; 1)
Phillip Kraft (William Beaumont Hospital, Troy; 14)
Tom Lassar (University Hospital Case Medical Center, Cleveland; 2)
Terrence Sacchi (New York Methodist Hospital, Brooklyn; 3)
Hani Kozman (SUNY Upstate Medical Univ, Syracuse; 6)
Rakesh Prashad (Ocala Research Institute, Ocala; 4)
Gregory San (Upstate Cardiology PA, Greenville; 11)
Augusto Villa (Advanced Clinical Research Associates, Jupiter; 5)
David Drenning (Huntsville Hospital, Huntsville; 29)
Robert Applegat (Wake Forest University Health Sciences, Winston-Salem; 9)
Paul Hermany (Buxmont Medical Associates, Sellersville; 4)
Ronald Karlsberg (Access Clinical Trials, Beverly Hills; 2)
Michele Degregorio (St. Joseph Mercy Oakland Hospital, Pontiac; 12)
Richard Bach (Washington Univ School of Medicine, St Louis; 9)
Gregory Giugliano (Baystate Medical Center, Springfield; 5)
Bruce Graham (Medical Consultants P.C., Muncie; 19)
William Bowden (Santa Rosa Memorial Hosp, Healdsburg; 2)
Michael Rosenberg, Parag Patel (Michael Rosenberg, Park Ridge; 1)
Emmanouil Brilakis (Dallas VA Medical Center, Dallas; 10)
Sarah Fenton (Cardiology Associates of Green Bay, Ltd, Green Bay ; 11)
John Gassler (University of Rochester, Rochester; 20)
James Johnson, Jr (North MS Medical Center, Tupelo; 11)
Brent Mclaurin (AnMed Health, Anderson; 2)
Ali Sonel (Pittsburgh Veterans Healthcare System, Pittsburgh; 4)
Anil Chhabra (Cardiovascular Research, LLC,; 10)
Frans Vossenberg (Mary Washington Hospital, Fredericksburg; 3)
Phillip Horwitz (University of Iowa Hospital Clinics, Iowa City; 14)
Kenneth Baran (St. Paul Heart Clinic, St Paul; 9)
Ellis Lader (Mid Valley Cardiology, Kingston; 3)
Adrian Magee (Inova/Fairfax Hospital Inst of Research Education, Falls Church; 1)
Jeffrey Chambers (Metropolitan Cardiology Consultants; 15)
James Delemos, Tayo Addo (Parkland Memorial Hospital, Dallas; 7)
George Fehrenbacher (Sutter Roseville Family Prac, Roseville; 2)
Paul Grossman (University of Michigan Medical Center, Ann Arbor; 4)
Kishore Harjai (Guthrie Clinic, Ltd., Sayre; 7)
Nasiruddin Jamal (Wilson Regional Medical Center / United Health Services Hospital, Johnson City; 1)
Abdulhay Albirini (Genesis HealthCare System, Zanesville; 12)
Violet Atanasoski-Mccormack (Broward General Medical Center, Ft Lauderdale; 26)
Thomas Ayres (Knoxville Heart Group, Knoxville; 10)
Kul Aggarwal (University of Missouri Hospitals & Clinic, Colombia; 23)
David Chang Moffitt Heart & Vascular Group, Wormleysburg; 2)

Ajay Labroo (Trinity Medical Center, Rock Island; 1)
Vance Wilson (Cardiology Consultants, Daytona Beach; 1)
Luis Rodriguez-Ospina (VA Caribbean Healthcare System, San Juan; 8)
Ryan Whitney (Bryan LGH Hospital, Lincoln; 7)
John Lopez, Dr Sandeep Nathan (University of Chicago Hospital, Chicago; 3)
Gary Schaer (Rush University Medical Center, Chicago; 4)
Barry Weinstock (Orlando Regional Medical Center, Orlando; 3)
Nicholaos Xenopoulos (Jewish Hospital Healthcare Inc, Louisville; 1)
Frank Zidar (Austin Heart P.A., Austin; 42)
James Blankenship (Geisinger Clinic, Danville; 2)
Cary Hirsch, Marcus Williams (Valley Hospital, Oakland; 5)
Frank Mcgrew (Baptist Memorial Hospital Health Care, Memphis; 4)
Nandkishore Ranadive (Florida Hospital, Orlando; 7)
Bernard Reen, Robert Iwaoka (Mid Carolina Cardiology, Charlotte; 45)
Robert Iwaoka (Trinity Medical Center, Rock Island; 5)
Jan Skowronski (Rockford Cardiology Research Foundation, Rockford; 2)
Gervasio Lamas (Mt. Sinai Medical Center, Miami Beach; 2)
Keith Rice (Mofitt Heart&Vascular Group - Holy Spirit Hospital Group, Wormsleyburg; 2)
Venkata Yalamanchili (Rudd Heart and Lung Center University of Louisville, Louisville; 4)
Robert Touchon (Kentucky Heart Institute, Ashland; 10)
Hal Wasserman (Regional Heart Vascular Center at Danbury Hospital, Danbury; 2)
Edo Kaluski (University of Medicine and Dentistry of NJ, Newark; 9)
Richard Lowry (Cardiovascular Associates of East Texas, Tyler; 7)
Thomas Mathew (Faxton-St. Lukes Health Care; 4)
Darshak Karia (Albert Einstein Medical Center, Philadelphia; 1)
Maher Rabah (William Beaumont Hospital, Royal Oak; 8)
M. Reza Movahed (University of Arizona Sarver Heart Center, Tucson; 3)
Mark Ricciardi (University Heakth Sciences Center, Albuquerque; 1)
Michael Foster (Sisters of Charity Providence Hospital, Columbia; 1)
Daniel Eisenberg (Foothill Cardiology California Heart Medical Group, Burbank; 1)
Tim Fischell (Borgess Research Institute, Kalamazoo; 1)
Johannes Brechtken, Shailesh Shetty (Regions Hospital St Pauls, St Pauls; 2)
James Trippi (Methodist Research Institute, Indianapolis; 4)
Joseph Rossi, Mauricio Cohen (UNC Chapel Hill, Chapel Hill; 2)
Joel Cohn (Thoracic and Cardiovascular Institute HeartCare, Lansing; 1)
Dan Fintel (Northwestern Hospital, Chicago; 3)
Luis Tami (Memorial Regional Hospital, Hollywood; 3)
Edward O'Leary (Nebraska Medical Center University Hospital, Omaha; 1)
David Safley (Saint Lukes Hospital, Kansas City; 3)
Thomas Stuckey (LeBauer CV Research Foundation, Greensboro; 19)
Atul Aggarwal (Nebraska Heart Institute, Lincoln; 2)

Data S1. Supplemental Methods

Description of spline models

In order to visualize the biomarker dynamics during the index hospitalization in myocardial infarction (MI) with non-obstructive coronary arteries (MINOCA) and with obstructive coronary artery disease (MI-CAD), linear regression models including time from symptom onset were fitted for each biomarker (high sensitivity cardiac troponin T [hs-cTnT], N-terminal pro-B-type natriuretic peptide [NT-proBNP], high sensitivity C-reactive protein [hs-CRP] and growth differentiation factor 15 [GDF-15]) as outcome variable. Time was modelled as a restricted cubic spline, with four knots placed at the 5th, 35th, 65th and 95th sample percentiles, to allow for non-linear associations and included MINOCA/MI-CAD classification and MI type (ST-elevation myocardial infarction [STEMI] or non-ST-elevation myocardial infarction [NSTEMI]) as well as all pairwise interactions among these three variables. To save degrees of freedom and avoid overfitting, the non-linearity interactions with time were done only with the linear part of the spline representation. Since concentrations typically are on a multiplicative rather than an additive scale, natural logarithmic transformations were applied for the biomarkers. However, the results were back-transformed to the original scale and the estimations from the models were consequently presented as geometric means. The p-value refers to a global test of the hypothesis of no interaction. That is, if the model with the three pairwise interactions of time, MINOCA/MI-CAD classification and MI type (STEMI/NSTEMI) was significantly better than a model without the interactions.

Furthermore, the models were adjusted for baseline clinical characteristics (age, sex, body mass index, current smoking, hypertension, dyslipidemia [medical history and/or lipid treatment at admission], diabetes mellitus, chronic kidney disease [medical history], previous MI, previous ischemic stroke, previous heart failure and peripheral vascular disease). Hence, the geometric mean displayed in the figure is for an example MI patient with the specified adjusted variables; categorical variables are set to the most frequent category (i.e. sex male) and continuous variables are set to the median (i.e. age 61 years).

Table S1. Medications at blood sampling one month after the index event in MINOCA and MI-CAD patients.

	Follow-up cohort at one month	
**Medications at blood sampling**	**MINOCA (n=107)**	**MI-CAD (n=2755)**
Aspirin	105 (98.1%)	2720 (98.7%)
Betablockers	90 (84.1%)	2460 (89.3%)
RAAS inhibitors	85 (79.4%)	2467 (89.5%)
Statins	95 (88.8%)	2645 (96.0%)
Calcium inhibitors	28 (26.2%)	553 (20.1%)

Data presented as numbers (with percentages). According to the study protocol, all patients were randomized to receive either ticagrelor or clopidogrel.

MI-CAD: myocardial infarction with obstructive coronary artery disease; MINOCA: myocardial infarction with non-obstructive coronary arteries; RAAS: renin-angiotensin-aldosterone system.

Figure S1. Empirical cumulative distribution function plots for biomarker concentrations at baseline in the total study cohort.

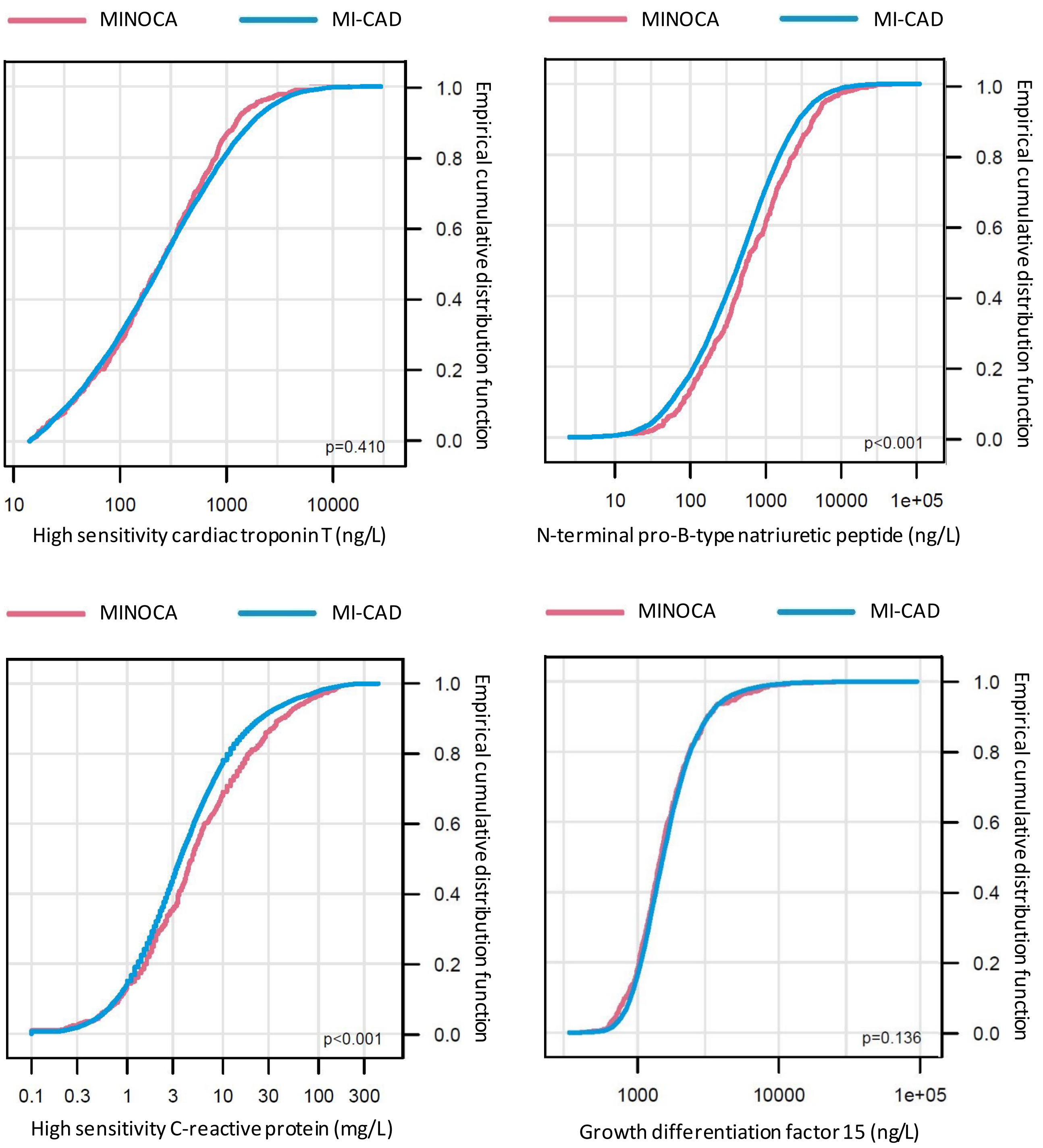

P-values are from Mann-Whitney tests of differences in biomarker concentration distributions between MINOCA and MI-CAD patients.

MI-CAD: myocardial infarction with obstructive coronary artery disease; MINOCA: myocardial infarction with non-obstructive coronary arteries.

Figure S2. Empirical cumulative distribution function plots for biomarker concentrations in the follow-up cohort at one month after the index MI.

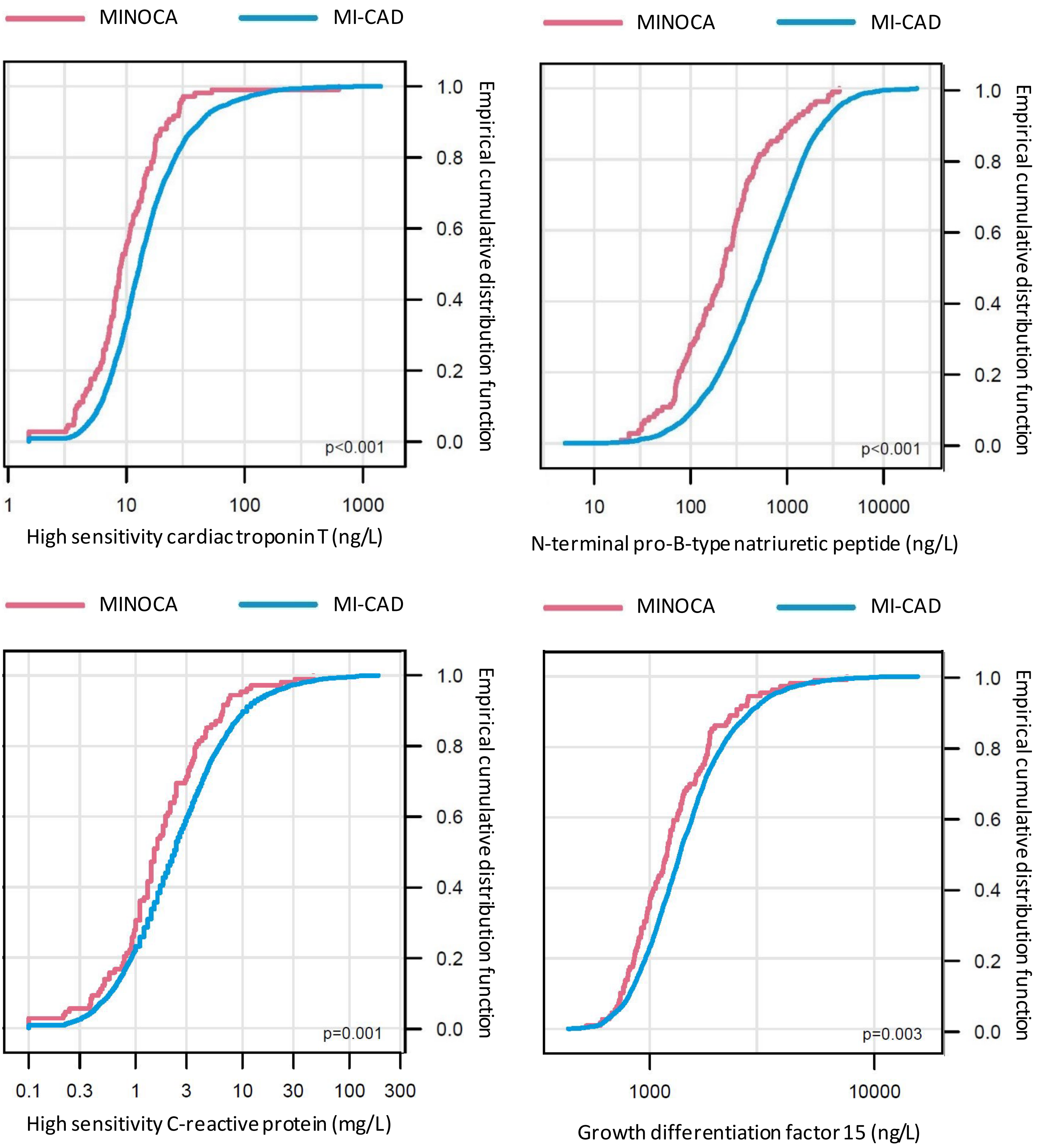

P-values are from Mann-Whitney tests of differences in biomarker concentration distributions between MINOCA and MI-CAD patients.

MI: myocardial infarction; MI-CAD: myocardial infarction with obstructive coronary artery disease; MINOCA: myocardial infarction with non-obstructive coronary arteries.
